# Uric Acid Mediated the Association Between BMI and Postmenopausal Breast Cancer Incidence: A Bidirectional Mendelian Randomization Analysis and Prospective Cohort Study

**DOI:** 10.3389/fendo.2021.742411

**Published:** 2022-02-03

**Authors:** Yue Feng, Ming Fu, Xin Guan, Chenming Wang, Fangfang Yuan, Yansen Bai, Hua Meng, Guyanan Li, Wei Wei, Hang Li, Mengying Li, Jiali Jie, Yanjun Lu, Huan Guo

**Affiliations:** ^1^ Department of Occupational and Environmental Health, Key Laboratory of Environment and Health, Ministry of Education and State Key Laboratory of Environmental Health (Incubating), School of Public Health, Tongji Medical College, Huazhong University of Science and Technology, Wuhan, China; ^2^ Department of Laboratory Medicine, Tongji Hospital, Tongji Medical College, Huazhong University of Science and Technology, Wuhan, China

**Keywords:** postmenopausal breast cancer, body mass index, uric acid, Mendelian randomization analysis, cohort study, mediation analysis

## Abstract

**Background:**

Observational epidemiological studies have reported the associations of high body mass index (BMI) with elevated serum uric acid (UA) level and increased risk of postmenopausal breast cancer. However, whether UA is causally induced by BMI and functioned in the BMI–breast cancer relationship remains unclear.

**Methods:**

To elucidate the causality direction between BMI and serum UA, the bidirectional Mendelian randomization (MR) analyses were performed by using summarized data from the largest Asian genome-wide association studies (GWAS) of BMI and UA carried out in over 150,000 Japanese populations. Then, a total of 19,518 postmenopausal women from the Dongfeng–Tongji (DFTJ) cohort (with a mean 8.2-year follow-up) were included and analyzed on the associations of BMI and serum UA with incidence risk of postmenopausal breast cancer by using multivariable Cox proportional hazard regression models. Mediation analysis was further conducted among DFTJ cohort to assess the intermediate role of serum UA in the BMI–breast cancer association.

**Results:**

In the bidirectional MR analyses, we observed that genetically determined BMI was causally associated with elevated serum UA [β(95% CI) = 0.225(0.111, 0.339), *p* < 0.001], but not vice versa. In the DFTJ cohort, each standard deviation (SD) increment in BMI (3.5 kg/m^2^) and UA (75.4 μmol/l) was associated with a separate 24% and 22% increased risk of postmenopausal breast cancer [HR(95% CI) = 1.24(1.07, 1.44) and 1.22(1.05, 1.42), respectively]. More importantly, serum UA could mediate 16.9% of the association between BMI and incident postmenopausal breast cancer.

**Conclusions:**

The current findings revealed a causal effect of BMI on increasing serum UA and highlighted the mediating role of UA in the BMI–breast cancer relationship. Controlling the serum level of UA among overweight postmenopausal women may help to decrease their incident risk of breast cancer.

## Introduction

Breast cancer is the most commonly diagnosed cancer (2.3 million, 11.7% of the total cancer cases) and the leading cause of cancer deaths among females (0.7 million, 15.0% of the total cancer deaths) (1). According to the latest cancer statistics of China in 2015, breast cancer accounts for 15.1% (268,600 new cases) of all new female cancers (2). The etiology of breast cancer differs between pre- and postmenopausal women because of the decreased ovarian hormone after menopause (3). As summarized in a meta-analysis of 34 prospective studies with more than 2.5 million females all over the world, higher body mass index (BMI) was observed to be associated with decreased breast cancer risk among premenopausal women but with increased breast cancer risk among postmenopausal women (4). Although pathways related to sex hormones and inflammation could partly explain the relationship between adiposity and breast cancer (5), exploring other potential biological intermediates may help better understand the underlying mechanisms.

Uric acid (UA) is the end oxidation product of purine metabolism in the human body, generated during enzymatic degradation of hypoxanthine and xanthine. A cross-sectional epidemiology study has reported a positive association between serum UA and BMI among 144,856 Chinese aged 20 to 79 years (6). Another longitudinal study among 2,611 young black and white adults revealed that baseline BMI was positively related to a 10-year change in serum UA (7). However, whether elevated serum UA is the cause or consequence of BMI is less investigated. Mendelian randomization (MR) is a useful method to explore the causality between a given exposure and outcome by using instrument variables (IVs) as proxies for exposure (8, 9). Single-nucleotide polymorphisms (SNPs) can be used as IVs since they are inherited randomly, and the MR approach using SNPs to predict phenotype is less prone to confounding and reverse causality than observational studies. Previous genome-wide association studies (GWAS) in large Japanese populations have identified plenty of SNPs associated with BMI and serum UA (10, 11). Treating these SNPs as IVs separately in the bidirectional MR analysis can help to test the direction of causation between BMI and UA.

UA was acknowledged to be a potent antioxidant in human plasma and might protect against cancer (12). Published epidemiological studies have investigated the hypothesis but provided conflicting findings. A meta-analysis of 5 independent cohort studies revealed that high serum UA was associated with an increased risk of total cancer incidence (13). Moreover, a Mendelian randomization study among 86,210 individuals from Copenhagen suggested the causal effect of high plasma urate on increased total cancer incidence risk (14). *In vitro* experiments revealed that UA lost its antioxidant ability in lipophilic conditions (15). Additionally, UA could increase the migratory rate of both human mammary cancer cells and mouse mammary epithelial cells, suggesting a potential link between UA and breast cancer (16). However, limited population-based studies have assessed the association between serum UA and breast cancer incidence risk and reported inconclusive results (17, 18), and no study yet has focused on the UA–postmenopausal breast cancer relationship. More importantly, whether serum UA may function as an intermediate link in BMI-postmenopausal breast cancer also remains to be clarified.

In the current study, we performed a bidirectional MR analysis to infer the causality direction between BMI and serum UA by using the summarized GWAS data of more than 150,000 Japanese populations. Then, we included 19,518 postmenopausal women from the prospective Dongfeng–Tongji (DFTJ) cohort, evaluated the associations of BMI and serum UA with incident risk of breast cancer, and explored the mediation effect of UA on the BMI–breast cancer relationship.

## Materials and Methods

### Bidirectional Mendelian Randomization Analysis

#### Study Population

**Figure 1:**
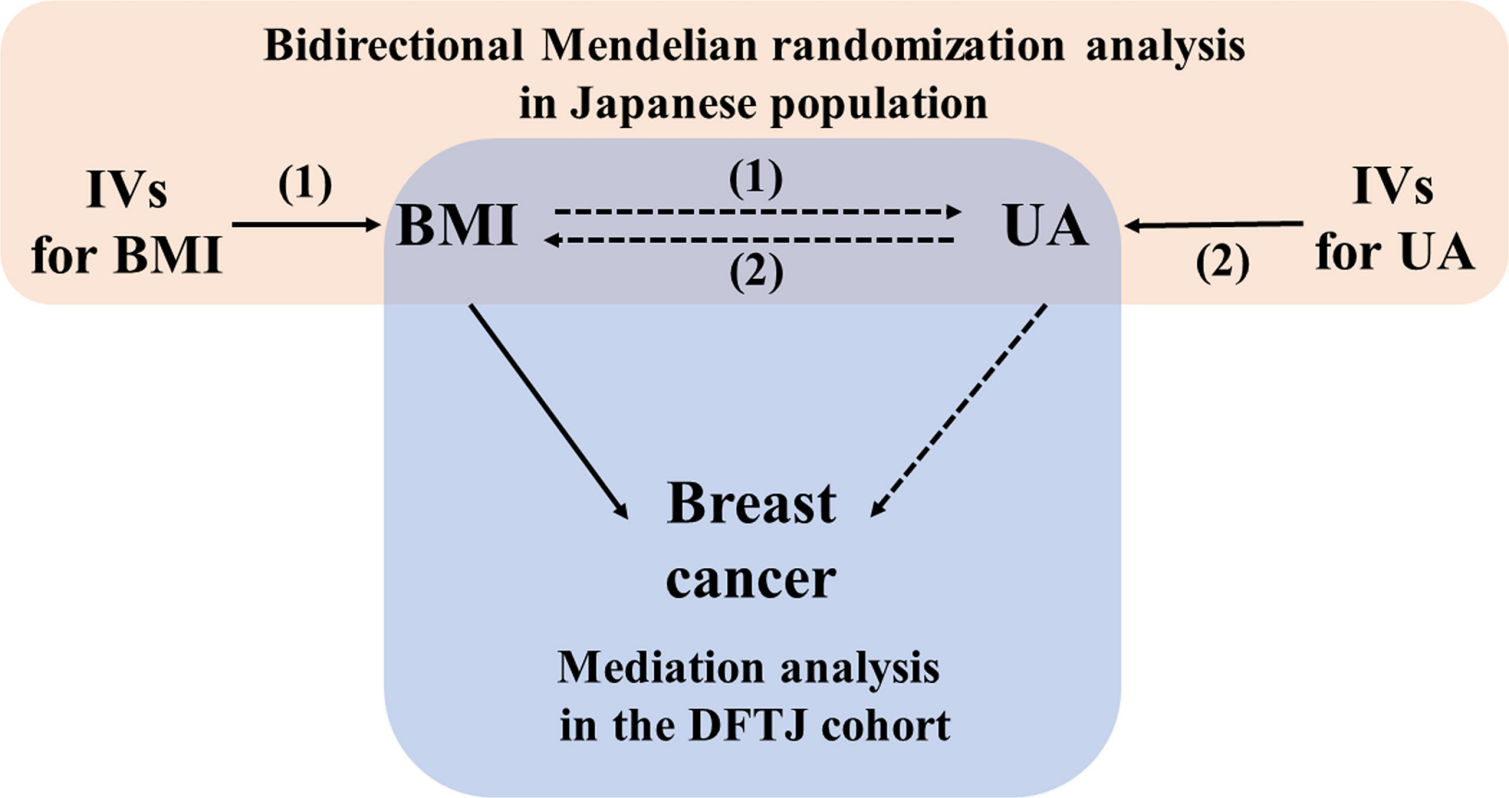
Schematic diagram of the study design. BMI, body mass index; DFTJ, Dongfeng Tongji; IV, instrument variable; UA, uric acid. (1): forward Mendelian randomization analysis (2); reverse Mendelian randomization analysis.

The study design is shown in **Figure 1**. The bidirectional MR analysis was based on summary-level data from the hitherto largest Asian GWAS. Summarized data were available from two studies with a total of more than 150,000 participants in 5 Japanese cohorts [including the BioBank Japan (BBJ) Project, the Japan Public Health Center-based Prospective Study (JPHC), the Tohoku Medical Megabank Project (TMM), the Japan Multi-institutional Collaborative Cohort (J-MICC) Study, and the Kita–Nagoya Genomic Epidemiology (KING) Study] (10, 11). Data were downloaded from the National Bioscience Database Center (NBDC) Human Database (https://humandbs.biosciencedbc.jp/en/).

#### Forward Mendelian Randomization Analysis

To test whether higher BMI is the cause of elevated serum UA, we first extracted the summary statistics for the BMI-related SNPs in the largest Asian GWAS among 173,430 Japanese participants (including 158,284 in the BBJ Project and 15,146 in the JPHC and the TMM Project) (10). This study used rank-based inverse-normal transformed BMI as the dependent variable and identified 83 independent SNPs which were significantly associated with BMI at *p* < 5 × 10^-8^. Then, the effect estimates [β and standard error (SE)] for the associations of these SNPs with UA were derived from a genome-wide meta-analysis based on 3 Japanese cohorts (n = 121,745), including 10,621 participants in the J-MICC Study, 2,095 participants in the KING Study, and 109,029 participants in the BBJ Project (11). After excluding 7 SNPs with missing estimates in the UA GWAS dataset, the left 76 BMI-associated SNPs were included as the candidate IVs in the forward MR analysis (**Table S1**).

#### Reverse Mendelian Randomization Analysis

To test whether higher serum UA is the cause of elevated BMI, we extracted the summary statistics for the SNP-UA association from the genome-wide meta-analysis among 121,745 Japanese participants mentioned above (11). This study employed Z-score-transformed serum UA as the dependent variable and identified 36 independent SNPs with genome-wide significant association with UA (*p* < 5 × 10^-8^). Then, the effect estimates [β and SE] for the associations between these 36 SNPs and BMI were extracted from the results among 158,284 participants in the BBJ project (10) and included in the reverse MR analysis (**Table S3**).

### Prospective Dongfeng–Tongji Cohort Study

#### Study Population

The DFTJ cohort is an ongoing prospective study carried out in Shiyan, China. The general information of this cohort has been described previously (19). Briefly, we recruited 27,009 retired workers in the Dongfeng Motor Corporation (DMC) from September 2008 to June 2010. Additional 14,120 retired workers were recruited into this cohort from April to October 2013. Among the whole population (n = 41,129), we excluded the males (n = 18,533), females with previous histories of malignant tumors at baseline (n = 690), females with regular menstrual cycles at enrollment (n = 2,298), and females who were younger than 50 years old and had missing information of menopausal status (n = 17) or stopped menstrual cycles following unknown disease reasons (n = 73) at baseline. Finally, the left 19,518 postmenopausal women were included in the subsequent analyses. All individuals signed informed consents to participate in this study and this work was approved by the Ethics Committee of Tongji Medical College, Huazhong University of Science and Technology.

#### Assessment of Covariates

Face-to-face questionnaire interviews were carried out to collect information of demographic characteristics (e.g., age, sex, and education levels), lifestyles (e.g., smoking and alcohol drinking status), female reproductive history (e.g., records of menopause, pregnancy, delivery, abortion, and contraception), and medication history [e.g., diuretics, antibiotics, and hormone replacement therapy (HRT) use]. Participants who smoked at least one cigarette per day for more than half a year were defined as current smokers, and those who ever smoked and had quitted over half a year were defined as former smokers; otherwise, they were defined as never smokers. Similarly, those who had drunk alcohol more than once a week for at least half a year were defined as current alcohol drinkers, and those who had ever drunk but quitted over half a year were defined as former alcohol drinkers; otherwise, they were defined as never drinkers. Both former and current smokers/alcohol drinkers were classified as ever smokers/alcohol drinkers. Marital status was collected as married, unmarried, separated, divorced, and widowed, then grouped into married or single status. Menopause was defined retrospectively as the cessation of menstrual cycles for 12 months occurring spontaneously, which was self-reported at baseline interview. Females with missing menopausal information and females who had stopped menstrual cycles due to disease reasons (all aged 50 years or older) were considered as postmenopausal in the present study. During the physical examination at enrollment, the anthropometric indicators (height, weight, and waist circumference) were measured with participants in light indoor clothing and without shoes or hats. BMI was calculated as weight (kilogram) divided by height (meter) squared (kg/m^2^).

For each participant, 5 ml peripheral venous blood was collected after overnight fasting into an ethylenediaminetetraacetic acid anticoagulant tube. The serum level of UA was determined by using the enzymatic uricase method. Briefly, UA was oxidized to allantoin and hydrogen peroxide by uricase. In the presence of hydrogen peroxide, a colored product was produced by the reaction of 4-aminoantipyrine with 3,5-dichloro-2-hydroxybenzene sulfonate, and the absorbance of such product was measured at 520 nm. All measurements were performed by experienced technicians using an ARCHITECT ci8200 automatic analyzer (Abbott Laboratories. Abbott Park, Illinois, USA) in the laboratory of Sinopharm Dongfeng General Hospital.

#### Ascertainment of Incident Breast Cancer

During the follow-up period, the new incident cases of breast cancer and dates of cancer diagnosis were confirmed by reviewing their medical records or death certificates in DMC’s healthcare system, which includes five DMC-owned hospitals and the local Center for Disease Control and Prevention. International Classification of Diseases, 10th Revision (ICD-10), was used to classify the incident breast cancer cases (ICD codes C50.000–C50.900). Among the whole 19,518 postmenopausal women, 211 new incident breast cancer cases were identified by December 31, 2018.

### Statistical Analysis

The bidirectional MR analysis used the summary-level data from two Japanese GWAS to infer the causal direction of association between BMI and serum UA. For forward MR analysis, we considered BMI as the exposure and serum UA as the outcome, and the BMI-related SNPs were used as IVs. In the reverse MR analysis, serum UA was considered as exposure and BMI was considered as the outcome, and the UA-related SNPs were used as IVs. MR analysis relies on 3 presuppositions: (1) the selected IVs are strongly associated with the exposure; (2) the IVs are not related to confounders for the exposure–outcome association; (3) IVs only affect the outcome through its effect on exposure. To test the assumption (1), we calculated the F-statistic value for each SNP and excluded weak IVs with F-value < 10. For assumptions (2) and (3), IVs with significant associations with diseases or traits other than the exposure of interest were excluded by searching PhenoScanner, a curated database of human genotype–phenotype associations (20). The MR analysis was mainly conducted by using the inverse-variance weighted (IVW) method in a fixed-effect meta-analysis model (21), and the sensitivity analyses by using weighted median method (22) and Mendelian Randomization Pleiotropy Residual Sum and Outlier (MR-PRESSO) method (23) were further conducted to test the robustness of associations. The weighted median method is based on the weighted median effect of all MR estimates produced by individual IV, with weight equal to the inverse of the standard error. MR-PRESSO can exclude outliers determined by the square of residual errors from the IV–outcome against IV–exposure regression to calculate an outlier-free effect estimate. The MR analyses were performed by “MendelianRandomization” R package.

For the female participants in the DFTJ cohort, their follow-up time was calculated from the date of enrollment to the date of cancer diagnosis, death, loss to follow-up, or end of the follow-up (December 31, 2018), whichever came first. We used multivariable Cox proportional hazard models to estimate the hazard ratios (HRs) and 95% confidence intervals (CIs) of breast cancer incidence risk associated with per standard deviation (SD) increment of BMI and serum UA. The proportional hazard assumption was examined by creating a product term of survival time and exposure, and we found no significant deviation from the assumption. We used three models to test the above associations: model 1 was adjusted for age (continuous), smoking and drinking status (ever/never), education level (middle school and below/high school and above), marital status (married/single), and batch to enter the cohort (2008/2013); model 2 further included female reproductive histories [parity (continuous), mastitis history (ever/never), age at menopause (continuous)], and medication use [diuretics, antibiotics, and HRT use (ever/never)] as the covariates; in model 3, the UA–breast cancer association was additionally adjusted for BMI and the BMI–breast cancer association was additionally adjusted for UA. Participants with missing information of exposure, outcome, or covariates were not included in the corresponding regression analyses. Besides, all participants were classified into four (Q1, Q2, Q3, and Q4) subgroups according to the quartiles of serum UA. When we used participants within the lowest UA quartile (Q1) as the reference group, the HRs and 95% CIs for the other three UA subgroups (Q2, Q3, and Q4) were calculated. To attenuate potential reverse causation, sensitivity analysis was performed by excluding participants diagnosed with breast cancer within the first year of follow-up. The association of waist circumference (another measurement of adiposity) with incident risk of postmenopausal breast cancer was also evaluated.

To further investigate the mediation effect of serum UA on the association between BMI and incident risk of postmenopausal breast cancer, causal mediation analysis was conducted for survival data within counterfactual framework by two statistical models (mediator model and outcome model) (24, 25). The mediator model referred to the generalized linear regression model for the association between BMI and serum level of UA (with adjustment for age, smoking and drinking status, education, batch to enter the cohort, marital status, parity, age at menopause, mastitis history, diuretics, antibiotics, and HRT use), and the outcome model referred to the Cox proportional hazard model for the association of BMI and serum UA with breast cancer incidence risk (including BMI, serum UA, and the above covariates). Mediation analyses with and without the exposure–mediator multiplicative interaction term (BMI × UA) were both performed and the estimates of direct and indirect effects did not change substantially (data not shown), so we did not further include the interaction term of BMI × UA in the outcome model (26). The natural indirect effect (NIE) is the effect of BMI on breast cancer mediated by UA, and natural direct effect (NDE) is the effect of BMI on breast cancer independent of UA, which can be estimated on the log hazard ratio scale. On the log hazard ratio scale, total effect (TE) can be decomposed into NIE and NDE: TE_log(HR)_ = NIE_log(HR)_ + NDE_log(HR)_, and the proportion mediated by UA can be calculated as NIE_log(HR)_/[NIE_log(HR)_ + NDE_log(HR)_]. Similarly, we explored the mediation role of serum UA on the association between waist circumference and breast cancer incidence risk. The mediation analysis was performed by “%mediation” SAS macro.

The statistical analyses were performed with the SAS program (version 9.4, SAS Institute, Carry, NC) and R software (version 3.6.3).

## Results

### Causal Associations Between BMI and Serum UA

For the forward MR analysis, all the 76 selected BMI-related SNPs had F-statistic values >10, suggesting that all candidate IVs for BMI were unlikely to introduce weak instrument bias into the MR analysis. The associations of these SNPs with BMI and UA are shown in **Table S1**. The IVW method by using 76 BMI-related SNPs as IVs revealed that the genetically predicted increase of BMI was causally associated with elevated serum UA [β (95% CI) = 0.183 (0.118, 0.248), *p* < 0.001] (**Table 1**). To attenuate the impact of pleiotropy, we excluded 44 SNPs significantly associated with traits other than BMI (**Table S2**), and the MR analysis by using the left 32 SNPs still yielded a significant causal effect of BMI on serum UA [IVW method, β (95% CI) = 0.225 (0.111, 0.339) and *p* < 0.001] (**Table 1**). Sensitivity analyses by using weighted median and MR-PRESSO methods also confirmed the causal association [β (95% CI) = 0.172 (0.072, 0.272) and 0.191 (0.105, 0.277), *p* = 0.001 and *p* < 0.001, respectively].

**Table 1:** Bidirectional mendelian randomization estimates for the casual associations between BMI and serum uric acid.

MR analysis	MR estimate β (95% CI)	*p*
**Forward MR (UA secondary to BMI)**		
* Using 76 reported SNPs as IVs*		
IVW method	0.183 (0.118, 0.248)	<0.001
Weighted median method	0.191 (0.129, 0.254)	<0.001
MR-PRESSO method	0.169 (0.120, 0.218)	<0.001
* Using 32 reported SNPs as IVs after excluding SNPs with pleiotropy*		
IVW method	0.225 (0.111, 0.339)	<0.001
Weighted median method	0.172 (0.072, 0.272)	0.001
MR-PRESSO method	0.191 (0.105, 0.277)	<0.001
**Reverse MR (BMI secondary to UA)**		
* Using 36 reported SNPs as IVs*		
IVW method	0.010 (-0.035, 0.054)	0.670
Weighted median method	-0.021 (-0.046, 0.005)	0.111
MR-PRESSO method	0.004 (-0.033, 0.041)	0.851
* Using 14 reported SNPs as IVs after excluding SNPs with pleiotropy*		
IVW method	-0.001 (-0.053, 0.051)	0.966
Weighted median method	-0.015 (-0.042, 0.012)	0.275
MR-PRESSO method	-0.018 (-0.049, 0.013)	0.296

BMI, body mass index; MR, Mendelian randomization; UA, uric acid.

In the reverse MR analysis, all 36 UA-related SNPs had F-statistic values > 10 and were included as IVs (**Table S3**). The genetically determined UA was not significantly associated with BMI by using IVW, weighted median, or MR-PRESSO methods (**Table 1**). After excluding 22 SNPs with pleiotropy (**Table S4**), we did not observe a significant association between genetically determined UA and BMI either [IVW method, β (95% CI) =-0.001 (-0.053, 0.051) and *p* = 0.966] (**Table 1**).

### General Characteristics for Postmenopausal Women in the DFTJ Cohort

A total of 211 incident postmenopausal breast cancer cases were documented during the mean follow-up of 8.2 years. The characteristics of study participants are presented in **Table 2**. The incident breast cancer cases were more likely to have a higher baseline weight, BMI, and waist circumference than non-cases (62.6 ± 9.5 kg vs. 59.5 ± 9.1 kg, 25.4 ± 3.8 kg/m^2^ vs. 24.3 ± 3.5 kg/m^2^, and 83.8 ± 9.4 cm vs. 81.4 ± 9.2 cm, respectively). The baseline serum level of UA was higher in incident breast cancer cases than in non-cases (282.5 ± 77.5 μmol/l vs. 273.1 ± 75.4 μmol/l).

**Table 2:** Baseline characteristics of the study population (n = 19,518).

Characteristics	Breast cancer (n = 211)	Non-cancer (n = 19,307)
Age (years, mean ± SD)	61.8 ± 7.9	61.3 ± 8.0
Height (cm, mean ± SD)	156.9 ± 5.4	156.3 ± 5.8
Weight (kg, mean ± SD)	62.6 ± 9.5	59.5 ± 9.1
BMI		
Continuous (kg/m^2^, mean ± SD)	25.4 ± 3.8	24.3 ± 3.5
<18.5	1 (0.5%)	504 (2.6%)
18.5–23.9	75 (35.5%)	8,628 (44.7%)
24–27.9	95 (45.0%)	6,728 (34.8%)
≥28	35 (16.6%)	2,602 (13.5%)
Missing	5 (2.4%)	845 (4.4%)
Waist (cm, mean ± SD)		
Continuous (cm, mean ± SD)	83.8 ± 9.4	81.4 ± 9.2
<80	66 (31.3%)	8,097 (41.9%)
≥80	140 (66.4%)	10,320 (53.5%)
Missing	5 (2.4%)	890 (4.6%)
Drinking, n (%)		
Current	13 (6.2%)	1441 (7.5%)
Former	3 (1.4%)	222 (1.2%)
Never	195 (92.4%)	17,612 (91.2%)
Missing	0	32 (0.2%)
Smoking, n (%)		
Current	4 (1.9%)	429 (2.2%)
Former	3 (1.4%)	170 (0.9%)
Never	203 (96.2%)	18,560 (96.1%)
Missing	1 (0.5%)	148 (0.8%)
Marriage, n (%)		
Married	180 (85.3%)	16,481 (85.4%)
Single	31 (14.7%)	2,764 (14.3%)
Missing	0	62 (0.3%)
Education, n (%)		
Middle school and below	125 (59.2%)	11,865 (61.5%)
High school and above	82 (38.9%)	7,281 (37.7%)
Missing	4 (1.9%)	161 (0.8%)
Parity, median (25^th^, 75^th^)	2 (1, 3)	2 (1, 3)
No. of abortions, median (25^th^, 75^th^)	1 (0, 2)	1 (0, 2)
No. of induced abortions, median (25^th^, 75^th^)	1 (0, 2)	1 (0, 2)
Contraception, n (%)		
No	157 (73.7%)	14,855 (76.9%)
Yes	56 (26.3%)	4,108 (21.3%)
Missing	0	344 (1.8%)
Contraception duration (years, mean ± SD)	11.7 ± 8.9	12.2 ± 8.2
First contraception age (years, mean ± SD)	28.9 ± 4.3	29.4 ± 4.5
Menopause age (years, mean ± SD)	49.8 ± 3.7	49.1 ± 3.7
HRT use, n (%)		
No	202 (95.7%)	18,295 (94.8%)
Yes	9 (4.3%)	598 (3.1%)
Missing	0	414 (2.1%)
Mastitis history, n (%)		
No	165 (78.2%)	15,192 (78.7%)
Yes	18 (8.5%)	884 (4.6%)
Missing	28 (13.3%)	3,231 (16.7%)
Antibiotics use, n (%)		
No	201 (95.3%)	17,632 (91.3%)
Yes	10 (4.7%)	1,675 (8.7%)
Diuretics use, n (%)		
No	207 (98.1%)	18,938 (98.1%)
Yes	4 (1.9%)	369 (1.9%)
Uric acid (μmol/L, mean ± SD)	282.5 ± 77.5	273.1 ± 75.4

BMI, body mass index; HRT, hormone replacement therapy; SD, standard deviation.

Values were shown as means ± SD, n (%), or median (25^th^, 75^th^).

### Associations of BMI and Serum UA With Incident Risk of Postmenopausal Breast Cancer

As shown in **Table 3**, each SD increase in BMI (3.5 kg/m^2^) was associated with 29% elevated risk of breast cancer incidence [model 1, HR (95% CI) = 1.29 (1.14, 1.47), *p* < 0.001]. Further adjustment for female reproductive events (parity, age at menopause, and mastitis history) and medication histories (diuretics, antibiotics, and HRT use) also confirmed the above association [model 2, HR (95% CI) = 1.31 (1.14, 1.50), *p* < 0.001], and additional adjustment for UA slightly attenuated the effect [model 3, HR (95% CI) = 1.24 (1.07, 1.44), *p* = 0.004]. When considering BMI as a categorical variable, females with BMI ≥ 24 kg/m^2^ (overweight) had a significantly higher incident risk of postmenopausal breast cancer than those with BMI < 24 kg/m^2^ [model 3, HR (95% CI) = 1.41 (1.02, 1.96), *p* = 0.037]. In addition, we observed that each SD increment in waist circumference (9.2 cm) was associated with 22% elevated risk [HR (95% CI) = 1.22 (1.05, 1.43)] (**Table S5**).

**Table 3:** The associations of BMI and serum uric acid with incident risk of postmenopausal breast cancer.

Variables	Person-years	Model 1*^a^*		Model 2*^b^*		Model 3*^c^*		Sensitivity analysis*^d^*	
		HR (95% CI)	*p*	HR (95% CI)	*p*	HR (95% CI)	*p*	HR (95% CI)	*p*
BMI (kg/m^2^)									
<24	346.8/75,740.9	Ref		Ref		Ref		Ref	
≥24	546.9/79,542.4	1.63 (1.23, 2.18)	0.001	1.55 (1.13, 2.13)	0.007	1.41 (1.02, 1.96)	0.037	1.30 (0.93, 1.82)	0.125
Per SD	893.7/155,283.3	1.29 (1.14, 1.47)	<0.001	1.31 (1.14, 1.50)	<0.001	1.24 (1.07, 1.44)	0.004	1.20 (1.02, 1.40)	0.026
Serum UA (μmol/L)									
Q1 (5–223)	139.8/40,045.0	Ref		Ref		Ref		Ref	
Q2 (224–265)	208.9/39,135.8	1.28 (0.83, 1.97)	0.266	1.61 (0.98, 2.65)	0.061	1.53 (0.93, 2.52)	0.095	1.83 (1.09, 3.10)	0.024
Q3 (266–315)	256.9/39,006.8	1.48 (0.97, 2.25)	0.066	1.80 (1.11, 2.93)	0.018	1.66 (1.02, 2.71)	0.043	1.92 (1.14, 3.23)	0.014
Q4 (316–814)	274.8/36,748.8	1.64 (1.08, 2.50)	0.020	2.38 (1.48, 3.83)	<0.001	2.06 (1.27, 3.35)	0.003	2.24 (1.33, 3.78)	0.003
Per SD	880.4/154,936.4	1.15 (1.01, 1.32)	0.041	1.29 (1.11, 1.49)	0.001	1.22 (1.05, 1.42)	0.010	1.25 (1.07, 1.46)	0.006

BMI, body mass index; SD, standard deviation; UA, uric acid.

^a^Model 1: with adjustment for age, smoking status, drinking status, education, marriage status, and batch to enter the cohort.

^b^Model 2: further adjusted for parity, age at menopause, mastitis history, diuretics, antibiotics, and HRT use.

^c^Model 3: UA-breast cancer association was additionally adjusted for BMI and the BMI-breast cancer association additionally adjusted for UA.

^d^Excluding participants diagnosed with breast cancer in the first follow-up year, with the employment of the same covariates in Model 3.

After adjustment for the common confounders (age, smoking and drinking status, education level, marital status, and batch to enter the cohort), per SD increment in serum UA (75.4 μmol/l) was associated with 15% increased incident risk of postmenopausal breast cancer [model 1, HR (95% CI) = 1.15 (1.01, 1.32), *p* = 0.041]. Further adjustment for female reproductive events and medication histories also revealed the above association [model 2, HR (95% CI) =1.29 (1.11, 1.49), *p* = 0.001], while additional adjustment for BMI slightly reduced the effect [model 3, HR (95% CI) = 1.22 (1.05, 1.42), *p* = 0.010]. When classifying participants into four subgroups (Q1 to Q4) according to quartiles of serum UA and considering participants within the lowest UA subgroup (Q1, <224 μmol/l) as the reference, those within Q3 (266–315 μmol/l) and Q4 (≥316 μmol/l) UA subgroups showed a significantly elevated incident risk of breast cancer [HR (95% CI) = 1.66 (1.02, 2.71) and 2.06 (1.27, 3.35), respectively]. Subsequent sensitivity analysis by excluding breast cancer cases diagnosed during the first year of follow-up also confirmed the above positive associations (**Table 3**).

### Mediation Effect of Serum UA on BMI-Breast Cancer Association

**Figure 2:**
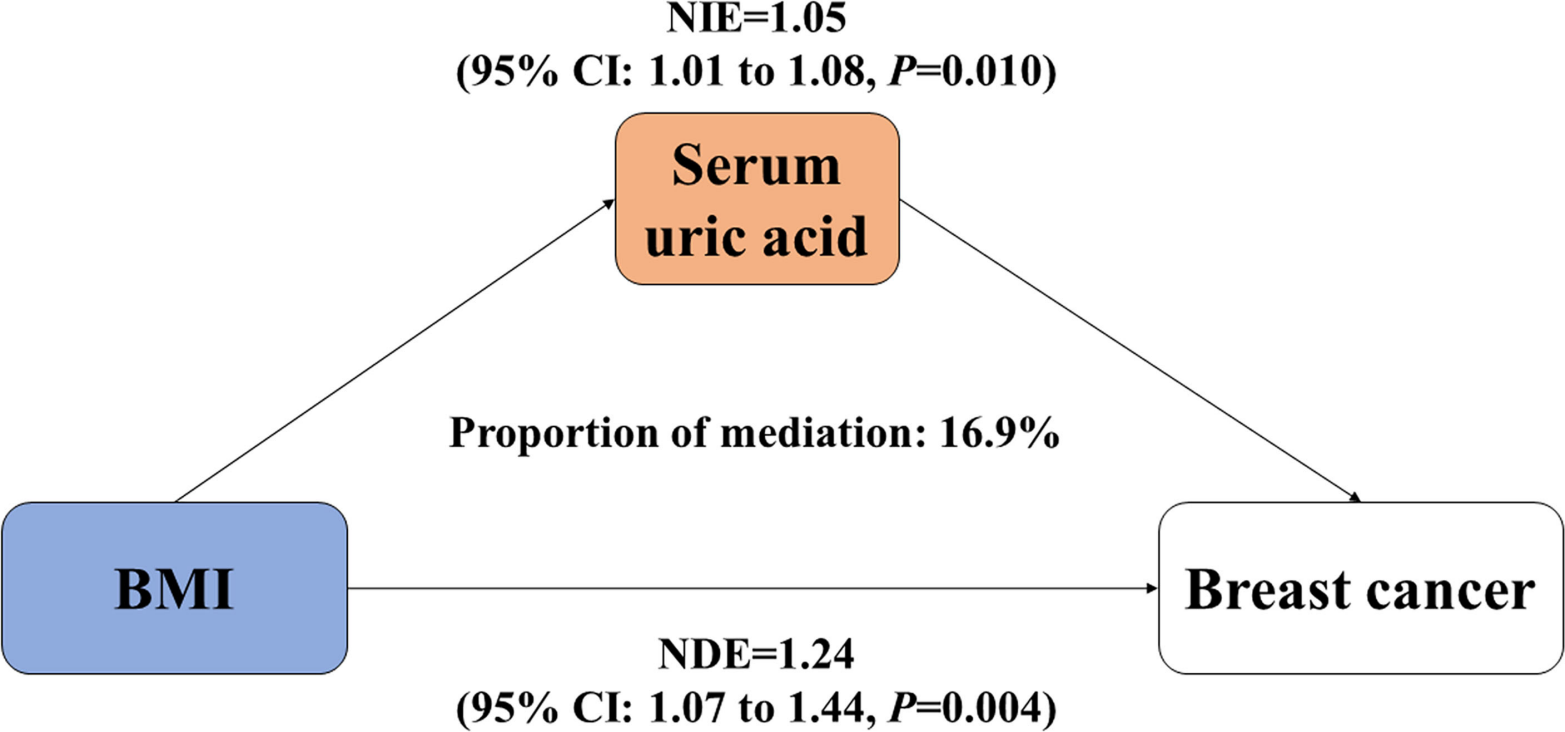
Mediation effect of serum uric acid on the association between BMI and incident risk of postmenopausal breast cancer. BMI, body mass index; NDE, natural direct effect; NIE, natural indirect effect. Both BMI and serum uric acid were treated as continuous variables.

Since increased BMI causally contributed to elevated serum UA, we treated UA as a mediator and further carried out the mediation analysis to explore the intermediate role of serum UA in BMI–breast cancer association. As shown in **Figure 2**, there were significant direct and indirect effects between BMI and incident risk of postmenopausal breast cancer [NDE = 1.24 (1.07, 1.44), *p* = 0.004, and NIE = 1.05 (1.01, 1.08), *p* = 0.010], and serum UA mediated 16.9% of the above association. Meanwhile, we observed that serum UA mediated 17.2% of the association between waist circumference and breast cancer risk [NDE = 1.22 (1.05, 1.43), NIE = 1.04 (1.01, 1.07)] (**Figure S1**).

## Discussion

The bidirectional MR analysis by using the largest Asian GWAS in Japanese populations revealed a causal effect of higher BMI on elevated serum UA, but not vice versa. In the DFTJ cohort, we observed positive associations of BMI, waist circumference, and serum UA with increased incident risk of postmenopausal breast cancer. More importantly, serum UA functioned as a significant mediator in the adiposity–breast cancer relationship.

To our knowledge, only two European MR studies have investigated the causal association between BMI and UA (27, 28). An MR analysis among 110,347 Europeans from the Global Urate Genetics Consortium used 97 previously reported SNPs as proxies for BMI and observed that each 4.6-kg/m^2^ increment in genetically predicted BMI was associated with a 0.3-mg/dl increase [95% CI (0.25, 0.35) mg/dl; *p* = 1.6 × 10^-36^] in serum UA, but the reverse effect of UA on BMI was not explored in their study (27). Another one-sample bidirectional MR study among 6,184 Europeans employed 3 SNPs in adiposity genes as IVs (rs1121980 in *FTO*, rs2665272 in *MC4R*, and rs6755502 in *TMEM18*) and did not find the causal effect of BMI on UA. The reverse MR analysis using rs6855911 in *SLC2A9* as the proxy for UA yielded a null causal effect of UA on BMI, either (28). Our study explored the direction of causation between BMI and UA by using the bidirectional MR approach among large Asian populations and observed the significant causal effect of higher BMI on elevated serum UA. The basic assumptions of MR analysis were fulfilled in our study. First, SNPs selected as candidate IVs were significantly associated with exposure of interest (BMI or UA) with F-value >10 and *p* for associations <5 × 10^-8^. Second, these IVs were not directly associated with the outcome and SNPs related to other phenotypes were excluded in the sensitivity analyses. Thus, the selected IVs affected the outcome through their effect on exposure, making the observed causal effect robust. However, we could not perform the stratified study of men and women in the MR analysis because no stratified GWAS of BMI and UA were carried out by gender in the reported publications. We assessed the correlations between measured BMI and serum UA in females and males of the DFTJ cohort separately and observed similar correlations in both gender (r = 0.224 and 0.188, respectively, data not shown), which was consistent with the results reported by a study in Japanese twin adults (29). The association of increased BMI and elevating serum UA is biologically plausible. Adipose tissue of obese mice was reported to have higher xanthine oxidoreductase activities and secrete excess UA (30). Enormous appetite of overweight people and ingestions of abundant purine-rich food could result in overproduction of UA (31), and obesity might hinder kidney clearance of UA (32, 33) by inducing insulin resistance (IR) *via* c-Jun amino-terminal kinases activity (34). These findings postulate elevating UA as the consequence of increased BMI, but further animal or functional experiments are needed to reveal the underlying mechanisms.

In line with our findings, many epidemiological studies have reported the effect of adiposity on increased risk of postmenopausal breast cancer (4, 35). A meta-analysis of 31 prospective studies suggested that each 5-kg/m^2^ increase in BMI was associated with a 12% increased risk of postmenopausal breast cancer [RR (95% CI) = 1.12 (1.08, 1.16)]. The meta-regression analysis of 5 Asia-Pacific cohort studies also suggested a robust effect of BMI, with each 5-kg/m^2^ increment in BMI associated with 1.31-fold (95% CI = 1.15–1.48) risk of postmenopausal breast cancer (4), which was comparable to our findings. In the current DFTJ cohort, we observed a 31% increased risk of postmenopausal breast cancer along with a per 3.5-kg/m^2^ increase in BMI among Chinese women. As another adiposity measurement, the increased waist circumference was also reported to be associated with elevated risk of postmenopausal breast cancer in previous literatures (36, 37), and this finding was consistently observed in the current cohort. Additionally, we observed a positive association of serum UA with increased incident risk of breast cancer among postmenopausal females. A follow-up study among 228,482 Swedish individuals (mean ± SD for age were 43.2 ± 13.8) observed that participants with serum UA level ≥ 279 μmol/l had a 7% reduced incident risk of breast cancer than those with serum UA level < 207 μmol/l [HR (95% CI) = 0.93 (0.88, 0.98)] (17). However, they did not separate the incident breast cancer cases according to the menopausal status nor adjust for potential confounding effects of smoking, drinking status, and reproductive histories. Another case–cohort study within the EPIC-Heidelberg cohort found a marginally inverse association between serum UA and breast cancer risk [Quartile 4 vs. Quartile 1, HR (95% CI) = 0.72 (0.53, 0.99)], and they did not distinguish pre- or postmenopausal women either. Furthermore, women in the EPIC-Heidelberg cohort had a significantly lower serum level of UA than women in the current DFTJ cohort (mean ± SD: 15.5 ± 4.0 μmol/l vs. 273.1 ± 75.4 μmol/l) (18). A Chinese perspective study enrolled 12,134 hypertensive females who were randomly assigned to receive a double-blind treatment of either enalapril (n = 6,064) or enalapril–folic acid (n = 6,070) and documented a separate 10 and 6 incident breast cancer cases during the 4.5-year follow-up and did not observe the association between serum UA and incidence risk of breast cancer [HR (95% CI) = 1.11 (0.68, 1.79) and 1.13 (0.59, 2.19) in the enalapril group and enalapril-folic acid group, respectively] (38). This study ignored the menopausal status, and the study participants had a higher mean serum UA level than postmenopausal women in the DFTJ cohort (303.5 vs. 273.1 μmol/l), which might result from the different health status at baseline. In addition, the small sample size of incident breast cancer cases (n = 16) limited the statistical power to uncover the association. Since the etiology of breast cancer varies between pre- and postmenopausal women, the above conflicting findings may be partly due to different distributions of menopausal status of the study participants. The distinct age distribution, human race, health status, and serum level of UA may also partly account for the inconsistent results across these studies. However, we could not perform MR analyses of BMI or UA with postmenopausal breast cancer only, because there was no GWAS of postmenopausal BRCA reported in Asian populations yet. Larger population-based prospective studies and MR analysis in different ethnic groups are warranted to validate the effects of UA on breast cancer among pre- or postmenopausal specifically.

Mediation analysis is usually employed in causal inference to clarify the roles of biological factors in the exposure-disease pathway. In the current DFTJ cohort, serum UA was found to mediate about 17% of the associations of BMI and waist circumference with increasing incident risk of postmenopausal breast cancer. Confounding of the association between the mediator (UA) and outcome (postmenopausal breast cancer) can introduce bias in the observational mediation analyses as any confounders might be part of the total effect of the exposure on the outcome. Thus, we have adjusted for many potential confounders (e.g., age, smoking and drinking status, and diuretic use). Novel mediation analyses by using MR and multivariable MR, which need the GWAS of exposure, mediator, and outcome, will overcome the limitation of traditional mediation analysis. However, we could not perform such analyses because no GWAS of postmenopausal breast cancer in Asian ancestry was reported. Further mediation analyses within the multivariable MR framework will help to reveal the mediation role of UA in BMI–breast cancer associations. An *in vitro* experiment observed that exposure to 1.6~25 mg/dl UA significantly increased the migratory rate of both human mammary cancer cells and mouse mammary epithelial cells, which suggested a functional link between UA and breast cancer (16). UA could act as a water-soluble radical scavenger and antioxidant in hydrophilic conditions but could not eliminate ROS in lipophilic conditions (15). In addition, the oxidation of UA could originate free radical metabolites (39). Cell experiments in mature adipocytes indicated that UA could induce the nicotinamide adenine dinucleotide phosphate (NADPH) oxidase-dependent oxidative stress (40), which then participated in the malignant transformation of breast epithelial cells (41), as well as the proliferation (42) and invasion of human breast cancer cells (43, 44). The increased oxidative stress might partly explain the biological functions of UA on adiposity-related breast cancer (45), but the underlying mechanisms warrant deep investigations.

The bidirectional MR analysis benefited from the GWAS results of the largest Asian population in more than 150,000 Japanese, which provided powerful evidence on the causal effect of BMI on elevating serum UA. Additionally, we provided the first longitudinal evidence on the mediation effect of serum UA on the adiposity–postmenopausal breast cancer association. Nevertheless, several limitations should not be ignored. Firstly, the influence of dietary habits on serum UA was not evaluated. However, the blood sampling after overnight fasting, as well as the similar living environment of our study participants, could attenuate the impact of diets. Secondly, UA only explained part of the association between BMI and postmenopausal breast cancer; some other important factors like the levels of free estradiol (46, 47) and insulin (47) may also link adiposity with breast cancer, and their roles need further explorations. Thirdly, a relatively moderate number of incident cases of postmenopausal breast cancer was documented during the 8.2-year follow-up in the DFTJ cohort (n = 211); future larger cohort studies with longer follow-up periods were warranted to validate the current findings. Fourthly, the effect of serum UA on premenopausal breast cancer still required further investigations. Lastly, we were unable to prove the causal effects of BMI and UA on postmenopausal breast cancer risk or conduct novel mediation analyses by using MR and multivariable MR analyses. Further research within the MR framework will help to prove our findings.

## Conclusions

Our study revealed that higher BMI could causally induce an elevated level of serum UA, but not vice versa. We found the positive associations of BMI and serum UA with increased incident risk of postmenopausal breast cancer in the DFTJ cohort and further documented the serum UA as an important mediator in adiposity-related breast cancer among Chinese postmenopausal women. Our study suggested the utility of UA as a clinical target for breast cancer prevention. Public and clinical implications of reducing serum UA might help to decrease breast cancer incidence among overweight postmenopausal women.

## Data Availability Statement

The raw data supporting the conclusions of this article will be made available by the authors, without undue reservation.

## Ethics Statement

The studies involving human participants were reviewed and approved by the Ethics Committee of Tongji Medical College, Huazhong University of Science and Technology. The participants provided their written informed consent to participate in this study.

## Author Contributions

HG and YF conceived this study, analyzed the data, interpreted the findings, drafted and revised the manuscript; MF, XG, CW, FY, and YB revised the manuscript and contributed to the further related research; HM, GL, WW, HL, ML, JJ, and YL helped the data collection and commented on the manuscript. All authors contributed to the article and approved the submitted version.

## Funding

This work was supported by the National Natural Scientific Foundation of China (grant numbers: 81722038 and 81773398) and the National Key Research and Development Program of China (grant number: 2018YFC2000203) to HG.

## Conflict of Interest

The authors declare that the research was conducted in the absence of any commercial or financial relationships that could be construed as a potential conflict of interest.

## Publisher’s Note

All claims expressed in this article are solely those of the authors and do not necessarily represent those of their affiliated organizations, or those of the publisher, the editors and the reviewers. Any product that may be evaluated in this article, or claim that may be made by its manufacturer, is not guaranteed or endorsed by the publisher.

## Abbreviations

BBJ, BioBank Japan; BMI, body mass index; CI, confidence interval; DFTJ, Dongfeng Tongji; DMC, Dongfeng Motor Corporation; GWAS, genome-wide association study; HR, hazard ratio; HRT, hormone replacement therapy; ICD, International Classification of Diseases; IV, instrument variable; IVW, inverse-variance weighted; JPHC, Japan Public Health Center–based Study; J-MICC, Japan Multi-institutional Collaborative Cohort; KING, Kita–Nagoya Genomic Epidemiology; MR, Mendelian randomization; MR-PRESSO, Mendelian Randomization Pleiotropy Residual Sum and Outlier; NADPH, nicotinamide adenine dinucleotide phosphate; NBDC, National Bioscience Database Center; NDE, natural direct effect; NIE, natural indirect effect; ROS, reactive oxygen species; SD, standard deviation; SE, standard error; SNP: single-nucleotide polymorphism; TE, total effect; TMM, Tohoku Medical Megabank; UA, uric acid.

## References

[1] Bray F Ferlay J Soerjomataram I Siegel RL Torre LA Jemal A. Global Cancer Statistics 2018: Globocan Estimates of Incidence and Mortality Worldwide for 36 Cancers in 185 Countries. CA Cancer J Clin (2018) 68:394–424. doi: 10.3322/caac.21492 30207593

[2] Chen W Zheng R Baade PD Zhang S Zeng H Bray F. Cancer Statistics in China, 2015. CA Cancer J Clin (2016) 66:115–32. doi: 10.3322/caac.21338 26808342

[3] Lu LJ Anderson KE Grady JJ Kohen F Nagamani M. Decreased Ovarian Hormones During a Soya Diet: Implications for Breast Cancer Prevention. Cancer Res (2000) 60:4112–21. 10945618

[4] Renehan AG Tyson M Egger M Heller RF Zwahlen M. Body-Mass Index and Incidence of Cancer: A Systematic Review and Meta-Analysis of Prospective Observational Studies. Lancet (2008) 371:569–78. doi: 10.1016/s0140-6736(08)60269-x 18280327

[5] IARC. Absence of Excess Body Fatness. IARC Handb Cancer Prev (2018) 16:1–646.

[6] Liu DM Jiang LD Gan L Su Y Li F. Association Between Serum Uric Acid Level and Body Mass Index in Sex- and Age-Specific Groups in Southwestern China. Endocr Pract (2019) 25:438–45. doi: 10.4158/ep-2018-0426 30657365

[7] Rathmann W Haastert B Icks A Giani G Roseman JM. Ten-Year Change in Serum Uric Acid and Its Relation to Changes in Other Metabolic Risk Factors in Young Black and White Adults: The CARDIA Study. Eur J Epidemiol (2007) 22:439–45. doi: 10.1007/s10654-007-9132-3 17484024

[8] Lawlor DA Harbord RM Sterne JA Timpson N Davey Smith G. Mendelian Randomization: Using Genes as Instruments for Making Causal Inferences in Epidemiology. Stat Med (2008) 27:1133–63. doi: 10.1002/sim.3034 17886233

[9] Thanassoulis G O’Donnell CJ. Mendelian Randomization: Nature’s Randomized Trial in the Post-Genome Era. JAMA (2009) 301:2386–8. doi: 10.1001/jama.2009.812 PMC3457799 19509388

[10] Akiyama M Okada Y Kanai M Takahashi A Momozawa Y Ikeda M. Genome-Wide Association Study Identifies 112 New Loci for Body Mass Index in the Japanese Population. Nat Genet (2017) 49:1458–67. doi: 10.1038/ng.3951 28892062

[11] Nakatochi M Kanai M Nakayama A Hishida A Kawamura Y Ichihara S. Genome-Wide Meta-Analysis Identifies Multiple Novel Loci Associated With Serum Uric Acid Levels in Japanese Individuals. Commun Biol (2019) 2:115. doi: 10.1038/s42003-019-0339-0 30993211 PMC6453927

[12] Ames BN Cathcart R Schwiers E Hochstein P. Uric Acid Provides an Antioxidant Defense in Humans Against Oxidant- and Radical-Caused Aging and Cancer: A Hypothesis. Proc Natl Acad Sci USA (1981) 78:6858–62. doi: 10.1073/pnas.78.11.6858 PMC349151 6947260

[13] Yan S Zhang P Xu W Liu Y Wang B Jiang T. Serum Uric Acid Increases Risk of Cancer Incidence and Mortality: A Systematic Review and Meta-Analysis. Mediators Inflamm (2015) 2015:764250. doi: 10.1155/2015/764250 26504361 PMC4609511

[14] Kobylecki CJ Afzal S Nordestgaard BG. Plasma Urate, Cancer Incidence, and All-Cause Mortality: A Mendelian Randomization Study. Clin Chem (2017) 63:1151–60. doi: 10.1373/clinchem.2016.268185 28428355

[15] Muraoka S Miura T. Inhibition by Uric Acid of Free Radicals That Damage Biological Molecules. Pharmacol Toxicol (2003) 93:284–9. doi: 10.1111/j.1600-0773.2003.pto930606.x 14675462

[16] Fini MA Orchard-Webb D Kosmider B Amon JD Kelland R Shibao G. Migratory Activity of Human Breast Cancer Cells Is Modulated by Differential Expression of Xanthine Oxidoreductase. J Cell Biochem (2008) 105:1008–26. doi: 10.1002/jcb.21901 PMC2587521 18767115

[17] Yiu A Van Hemelrijck M Garmo H Holmberg L Malmström H Lambe M. Circulating Uric Acid Levels and Subsequent Development of Cancer in 493,281 Individuals: Findings From the AMORIS Study. Oncotarget (2017) 8:42332–42. doi: 10.18632/oncotarget.16198 PMC5522070 28418841

[18] Kühn T Sookthai D Graf ME Schübel R Freisling H Johnson T. Albumin, Bilirubin, Uric Acid and Cancer Risk: Results From a Prospective Population-Based Study. Br J Cancer (2017) 117:1572–9. doi: 10.1038/bjc.2017.313 PMC5680462 28898231

[19] Wang F Zhu J Yao P Li X He M Liu Y. Cohort Profile: The Dongfeng-Tongji Cohort Study of Retired Workers. Int J Epidemiol (2013) 42:731–40. doi: 10.1093/ije/dys053 22531126

[20] Staley JR Blackshaw J Kamat MA Ellis S Surendran P Sun BB. PhenoScanner: A Database of Human Genotype-Phenotype Associations. Bioinformatics (2016) 32:3207–9. doi: 10.1093/bioinformatics/btw373 PMC5048068 27318201

[21] Burgess S Butterworth A Thompson SG. Mendelian Randomization Analysis With Multiple Genetic Variants Using Summarized Data. Genet Epidemiol (2013) 37:658–65. doi: 10.1002/gepi.21758 PMC4377079 24114802

[22] Bowden J Davey Smith G Haycock PC Burgess S. Consistent Estimation in Mendelian Randomization With Some Invalid Instruments Using a Weighted Median Estimator. Genet Epidemiol (2016) 40:304–14. doi: 10.1002/gepi.21965 PMC4849733 27061298

[23] Verbanck M Chen CY Neale B Do R. Detection of Widespread Horizontal Pleiotropy in Causal Relationships Inferred From Mendelian Randomization Between Complex Traits and Diseases. Nat Genet (2018) 50:693–8. doi: 10.1038/s41588-018-0099-7 PMC6083837 29686387

[24] VanderWeele TJ. Causal Mediation Analysis With Survival Data. Epidemiology (2011) 22:582–5. doi: 10.1097/EDE.0b013e31821db37e PMC3109321 21642779

[25] Vanderweele TJ Vansteelandt S. Odds Ratios for Mediation Analysis for a Dichotomous Outcome. Am J Epidemiol (2010) 172:1339–48. doi: 10.1093/aje/kwq332 PMC2998205 21036955

[26] VanderWeele TJ. Explanation in Causal Inference: Methods for Mediation and Interaction. New York: Oxford Univ. Press (2015).

[27] Larsson SC Burgess S Michaëlsson K. Genetic Association Between Adiposity and Gout: A Mendelian Randomization Study. Rheumatol (Oxford) (2018) 57:2145–8. doi: 10.1093/rheumatology/key229 PMC6697177 30085130

[28] Lyngdoh T Vuistiner P Marques-Vidal P Rousson V Waeber G Vollenweider P. Serum Uric Acid and Adiposity: Deciphering Causality Using a Bidirectional Mendelian Randomization Approach. PloS One (2012) 7:e39321. doi: 10.1371/journal.pone.0039321 22723994 PMC3378571

[29] Tsushima Y Nishizawa H Tochino Y Nakatsuji H Sekimoto R Nagao H. Uric Acid Secretion From Adipose Tissue and Its Increase in Obesity. J Biol Chem (2013) 288:27138–49. doi: 10.1074/jbc.M113.485094 PMC3779712 23913681

[30] Arafat AM Weickert MO Adamidou A Otto B Perschel FH Spranger J. The Impact of Insulin-Independent, Glucagon-Induced Suppression of Total Ghrelin on Satiety in Obesity and Type 1 Diabetes Mellitus. J Clin Endocrinol Metab (2013) 98:4133–42. doi: 10.1210/jc.2013-1635 23966238

[31] Perez-Ruiz F Aniel-Quiroga MA Herrero-Beites AM Chinchilla SP Erauskin GG Merriman T. Renal Clearance of Uric Acid Is Linked to Insulin Resistance and Lower Excretion of Sodium in Gout Patients. Rheumatol Int (2015) 35:1519–24. doi: 10.1007/s00296-015-3242-0 25763991

[32] Yamashita S Matsuzawa Y Tokunaga K Fujioka S Tarui S. Studies on the Impaired Metabolism of Uric Acid in Obese Subjects: Marked Reduction of Renal Urate Excretion and Its Improvement by a Low-Calorie Diet. Int J Obes (1986) 10:255–64. 3771090

[33] Hirosumi J Tuncman G Chang L Görgün CZ Uysal KT Maeda K. A Central Role for JNK in Obesity and Insulin Resistance. Nature (2002) 420:333–6. doi: 10.1038/nature01137 12447443

[34] Neuhouser ML Aragaki AK Prentice RL Manson JE Chlebowski R Carty CL. Overweight, Obesity, and Postmenopausal Invasive Breast Cancer Risk: A Secondary Analysis of the Women’s Health Initiative Randomized Clinical Trials. JAMA Oncol (2015) 1:611–21. doi: 10.1001/jamaoncol.2015.1546 PMC5070941 26182172

[35] White AJ Nichols HB Bradshaw PT Sandler DP. Overall and Central Adiposity and Breast Cancer Risk in the Sister Study. Cancer (2015) 121:3700–8. doi: 10.1002/cncr.29552 PMC4592412 26193782

[36] Lee KR Hwang IC Han KD Jung J Seo MH. Waist Circumference and Risk of Breast Cancer in Korean Women: A Nationwide Cohort Study. Int J Cancer (2018) 142:1554–9. doi: 10.1002/ijc.31180 29193045

[37] Yang J Wang Y Zhao Q Zhang X Wang X Qin X. Association of Serum Uric Acid With Increased Risk of Cancer Among Hypertensive Chinese. Int J Cancer (2017) 141:112–20. doi: 10.1002/ijc.30731 28393356

[38] Maples KR Mason RP. Free Radical Metabolite of Uric Acid. J Biol Chem (1988) 263:1709–12. doi: 10.1016/S0021-9258(19)77933-2 2828349

[39] Sautin YY Nakagawa T Zharikov S Johnson RJ. Adverse Effects of the Classic Antioxidant Uric Acid in Adipocytes: NADPH Oxidase-Mediated Oxidative/Nitrosative Stress. Am J Physiol Cell Physiol (2007) 293:C584–96. doi: 10.1152/ajpcell.00600.2006 17428837

[40] Mohapatra P Preet R Das D Satapathy SR Siddharth S Choudhuri T. The Contribution of Heavy Metals in Cigarette Smoke Condensate to Malignant Transformation of Breast Epithelial Cells and *In Vivo* Initiation of Neoplasia Through Induction of a PI3K-AKT-Nfκb Cascade. Toxicol Appl Pharmacol (2014) 274:168–79. doi: 10.1016/j.taap.2013.09.028 24099783

[41] Ruiz-Ramos R Lopez-Carrillo L Rios-Perez AD De Vizcaya-Ruíz A Cebrian ME. Sodium Arsenite Induces Ros Generation, DNA Oxidative Damage, HO-1 and C-Myc Proteins, NF-kappaB Activation and Cell Proliferation in Human Breast Cancer MCF-7 Cells. Mutat Res (2009) 674:109–15. doi: 10.1016/j.mrgentox.2008.09.021 18996220

[42] Yamazaki S Miyoshi N Kawabata K Yasuda M Shimoi K. Quercetin-3-O-Glucuronide Inhibits Noradrenaline-Promoted Invasion of MDA-MB-231 Human Breast Cancer Cells by Blocking β₂-Adrenergic Signaling. Arch Biochem Biophys (2014) 557:18–27. doi: 10.1016/j.abb.2014.05.030 24929186

[43] Zhang JW Rubio V Zheng S Shi ZZ. Knockdown of OLA1, a Regulator of Oxidative Stress Response, Inhibits Motility and Invasion of Breast Cancer Cells. J Zhejiang Univ Sci B (2009) 10:796–804. doi: 10.1631/jzus.B0910009 19882753 PMC2772883

[44] Ambrosone CB. Oxidants and Antioxidants in Breast Cancer. Antioxid Redox Signaling (2000) 2:903–17. doi: 10.1089/ars.2000.2.4-903 11213491

[45] Dashti SG Simpson JA Karahalios A Viallon V Moreno-Betancur M Gurrin LC. Adiposity and Estrogen Receptor-Positive, Postmenopausal Breast Cancer Risk: Quantification of the Mediating Effects of Fasting Insulin and Free Estradiol. Int J Cancer (2020) 146:1541–52. doi: 10.1002/ijc.32504 31187481

[46] Schairer C Fuhrman BJ Boyd-Morin J Genkinger JM Gail MH Hoover RN. Quantifying the Role of Circulating Unconjugated Estradiol in Mediating the Body Mass Index-Breast Cancer Association. Cancer Epidemiol Biomarkers Prev (2016) 25:105–13. doi: 10.1158/1055-9965.Epi-15-0687 PMC5555590 26637268

[47] Hvidtfeldt UA Gunter MJ Lange T Chlebowski RT Lane D Farhat GN. Quantifying Mediating Effects of Endogenous Estrogen and Insulin in the Relation Between Obesity, Alcohol Consumption, and Breast Cancer. Cancer Epidemiol Biomarkers Prev (2012) 21:1203–12. doi: 10.1158/1055-9965.Epi-12-0310 PMC3858186 22564867

