# Supplementary material for: Uric Acid Mediated the Association Between BMI and Postmenopausal Breast Cancer Incidence: A Bidirectional Mendelian Randomization Analysis and Prospective Cohort Study
Source: Front Endocrinol (Lausanne). 2022 Feb 3;12:742411. doi: 10.3389/fendo.2021.742411 (PMC8850312; doi:10.3389/fendo.2021.742411)
Supplement: Supplementary file 1 [file DataSheet_1.docx]

**Supplementary Tables and Figures**

**Table S1.** Characteristic of 83 BMI-related SNPs and their associations with BMI and serum UA in published GWASs.

**Table S2.** Associations of 83 BMI-related SNPs with potential pleiotropic effects from the PhenoScanner database (*P* < 5×10^-8^).

**Table S3.** Characteristic of 36 UA-related SNPs and their associations with UA and BMI in published GWASs.

**Table S4.** Associations of 36 uric acid-related SNPs with potential pleiotropic effects from the PhenoScanner database (*P* < 5×10^-8^).

**Table S5.** The associations of waist circumference with incident risk of postmenopausal breast cancer.

**Figure S1.** Mediation effect of serum uric acid on the association between waist circumference and incident risk of postmenopausal breast cancer.

**Abbreviations:** NDE, natural direct effect; NIE, natural indirect effect.

**Note:** Both waist circumference and serum uric acid were treated as continuous variables.

| **Table S1. Characteristic of 83 BMI-related SNPs and their associations with BMI and serum UA in published GWAS.** | | | | | | | | | | | | | |
| --- | --- | --- | --- | --- | --- | --- | --- | --- | --- | --- | --- | --- | --- |
| **SNP** | **Chr** | **Position ^a^** | **Gene** | **Effect allele ^b^** | **EAF** | **Association with BMI ^c^** | | | |  | **Association with serum UA ^e^** | | |
|  |  |  |  |  |  | **β** | **SE** | ***P*** | **F ^d^** |  | **β** | **SE** | ***P*** |
| rs2076463 | 1 | 27971092 | *FGR, IFI6* | A | 0.736 | 0.023 | 0.004 | 1.68E-08 | 33.06 |  | 0.005 | 0.005 | 0.273 |
| rs860295 | 1 | 155767708 | *GON4L* | G | 0.267 | 0.029 | 0.004 | 8.66E-11 | 52.56 |  | 0.025 | 0.005 | 2.05E-06 |
| rs633715 | 1 | 177852580 | *LOC101928778, SEC16B* | C | 0.225 | 0.049 | 0.004 | 1.05E-33 | 150.06 |  | 0.006 | 0.005 | 0.238 |
| rs491055 | 1 | 190308834 | *BRINP3* | A | 0.370 | 0.022 | 0.004 | 2.82E-10 | 30.25 |  | 0.008 | 0.004 | 0.044 |
| rs939584 | 2 | 621558 | *FAM150B, TMEM18* | T | 0.897 | 0.056 | 0.006 | 6.40E-23 | 87.11 |  | 0.033 | 0.007 | 5.52E-07 |
| rs713586 | 2 | 25158008 | *ADCY3, DNAJC27* | C | 0.511 | 0.025 | 0.003 | 4.92E-13 | 69.44 |  | 0.008 | 0.004 | 0.039 |
| rs6734118 | 2 | 37559355 | *PRKD3, QPCT* | A | 0.539 | 0.024 | 0.003 | 3.42E-12 | 64.00 |  | -0.004 | 0.004 | 0.338 |
| rs77489951 | 2 | 38750287 | *LOC101929596, HNRNPLL* | T | 0.064 | 0.044 | 0.008 | 9.39E-09 | 30.25 |  | -0.014 | 0.009 | 0.107 |
| rs10174398 | 2 | 51195601 | *NRXN1* | T | 0.423 | 0.020 | 0.003 | 4.65E-09 | 44.44 |  | 0.006 | 0.004 | 0.141 |
| rs10208649 | 2 | 54161363 | *PSME4* | T | 0.987 | 0.111 | 0.016 | 4.95E-12 | 48.13 |  | -0.015 | 0.018 | 0.422 |
| rs10197655 | 2 | 58791420 | *LINC01122* | G | 0.401 | 0.020 | 0.003 | 3.63E-09 | 44.44 |  | 0.006 | 0.004 | 0.119 |
| rs12617004 | 2 | 142615136 | *LRP1B* | C | 0.393 | 0.020 | 0.004 | 5.83E-09 | 25.00 |  | 0.017 | 0.004 | 2.85E-05 |
| rs2390669 | 2 | 169091942 | *STK39* | C | 0.244 | 0.024 | 0.004 | 5.63E-10 | 36.00 |  | 0.004 | 0.005 | 0.364 |
| rs6433857 | 2 | 181517996 | *CWC22, SCHLAP1* | C | 0.685 | 0.021 | 0.004 | 4.57E-08 | 27.56 |  | 0.009 | 0.004 | 0.053 |
| rs2574704 | 3 | 11655381 | *VGLL4* | C | 0.649 | 0.019 | 0.004 | 4.70E-08 | 22.56 |  | -0.007 | 0.004 | 0.116 |
| rs8192473 | 3 | 42299399 | *CCK* | C | 0.903 | 0.035 | 0.006 | 3.58E-09 | 34.03 |  | 0.007 | 0.007 | 0.337 |
| rs11130319 | 3 | 52755592 | *NEK4* | T | 0.534 | 0.023 | 0.003 | 3.72E-11 | 58.78 |  | -0.018 | 0.004 | 4.70E-06 |
| rs4686392 | 3 | 185524081 | *IGF2BP2* | A | 0.663 | 0.032 | 0.004 | 1.42E-18 | 64.00 |  | 0.000 | 0.004 | 0.922 |
| rs1996023 | 4 | 45164637 | *GNPDA2, GABRG1* | T | 0.29 | 0.032 | 0.004 | 1.06E-17 | 64.00 |  | 0.009 | 0.004 | 0.052 |
| rs1035491 | 5 | 63962177 | *RGS7BP, FAM159B* | A | 0.759 | 0.024 | 0.004 | 5.63E-09 | 36.00 |  | 0.007 | 0.005 | 0.152 |
| rs6881648 | 5 | 74991849 | *POC5* | A | 0.440 | 0.024 | 0.003 | 2.03E-12 | 64.00 |  | 0.003 | 0.004 | 0.448 |
| rs1846974 | 5 | 87969927 | *LINC00461* | A | 0.491 | 0.027 | 0.003 | 1.81E-15 | 81.00 |  | -0.001 | 0.004 | 0.814 |
| rs10062657 | 5 | 95867908 | *PCSK1* | C | 0.406 | 0.038 | 0.004 | 3.89E-25 | 90.25 |  | 0.005 | 0.004 | 0.241 |
| rs4308481 | 5 | 122652106 | *PRDM6, CEP120* | C | 0.510 | 0.021 | 0.004 | 9.71E-09 | 27.56 |  | -- | -- | -- |
| rs4357030 | 5 | 124316031 | *ZNF608, LOC101927421* | T | 0.529 | 0.024 | 0.004 | 3.60E-10 | 36.00 |  | 0.009 | 0.004 | 0.048 |
| rs329120 | 5 | 133861756 | *JADE2* | C | 0.628 | 0.019 | 0.004 | 3.70E-08 | 22.56 |  | -0.002 | 0.004 | 0.602 |
| rs35261542 | 6 | 20675792 | *CDKAL1* | C | 0.571 | 0.039 | 0.004 | 3.61E-29 | 95.06 |  | 0.013 | 0.004 | 0.001 |
| rs183975233 | 6 | 32437160 | *HLA-DRA, HLA-DRB5* | T | 0.611 | 0.031 | 0.004 | 7.51E-16 | 60.06 |  | 0.015 | 0.005 | 0.001 |
| rs6913361 | 6 | 34179390 | *GRM4, HMGA1* | A | 0.148 | 0.038 | 0.005 | 2.73E-14 | 57.76 |  | 0.007 | 0.006 | 0.219 |
| rs2206271 | 6 | 50786008 | *TFAP2B* | A | 0.351 | 0.031 | 0.004 | 2.77E-18 | 60.06 |  | -0.005 | 0.004 | 0.208 |
| rs148546399 | 6 | 64705610 | *EYS* | A | 0.054 | 0.050 | 0.008 | 1.13E-09 | 39.06 |  | 0.008 | 0.010 | 0.402 |
| rs9397585 | 6 | 153396875 | *RGS17* | C | 0.646 | 0.021 | 0.004 | 5.05E-09 | 27.56 |  | 0.011 | 0.004 | 0.011 |
| rs6947395 | 7 | 69406661 | *AUTS2* | T | 0.198 | 0.029 | 0.004 | 4.81E-12 | 52.56 |  | 0.003 | 0.005 | 0.620 |
| rs143665886 | 7 | 115368366 | *LINC01392, TFEC* | C | 0.413 | 0.022 | 0.004 | 9.46E-09 | 30.25 |  | 0.005 | 0.005 | 0.318 |
| rs77636220 | 8 | 64552779 | *LOC102724612, LINC01289* | A | 0.204 | 0.028 | 0.004 | 2.80E-11 | 49.00 |  | 0.008 | 0.005 | 0.104 |
| rs28857569 | 8 | 76697034 | *HNF4G, LINC01111* | C | 0.315 | 0.023 | 0.004 | 2.00E-09 | 33.06 |  | 0.009 | 0.004 | 0.046 |
| rs4366055 | 8 | 95507328 | *KIAA1429* | A | 0.510 | 0.020 | 0.003 | 2.12E-09 | 44.44 |  | 0.001 | 0.004 | 0.878 |
| rs7020996 | 9 | 22129579 | *CDKN2B-AS1, DMRTA1* | T | 0.419 | 0.032 | 0.004 | 5.87E-18 | 64.00 |  | 0.010 | 0.004 | 0.024 |
| rs10868215 | 9 | 87234111 | *SLC28A3, NTRK2* | T | 0.679 | 0.021 | 0.004 | 1.34E-08 | 27.56 |  | 0.001 | 0.004 | 0.834 |
| rs3932549 | 9 | 97073588 | *ZNF169, NUTM2F* | C | 0.687 | 0.025 | 0.004 | 1.97E-09 | 39.06 |  | 0.001 | 0.005 | 0.849 |
| rs5015933 | 9 | 128137418 | *GAPVD1, MAPKAP1* | T | 0.460 | 0.021 | 0.003 | 6.12E-10 | 49.00 |  | 0.011 | 0.004 | 0.005 |
| rs10795945 | 10 | 12302607 | *CDC123, CAMK1D* | C | 0.557 | 0.021 | 0.003 | 1.10E-09 | 49.00 |  | 0.008 | 0.004 | 0.037 |
| rs7912454 | 10 | 18584792 | *CACNB2* | A | 0.830 | 0.029 | 0.005 | 3.62E-10 | 33.64 |  | 0.005 | 0.005 | 0.357 |
| rs80117551 | 10 | 69834828 | *HERC4* | C | 0.745 | 0.022 | 0.004 | 1.57E-08 | 30.25 |  | 0.008 | 0.005 | 0.107 |
| rs1832886 | 10 | 94477539 | *HHEX, EXOC6* | A | 0.784 | 0.031 | 0.004 | 2.59E-12 | 60.06 |  | 0.012 | 0.005 | 0.024 |
| rs12569457 | 10 | 99096676 | *FRAT2, RRP12* | T | 0.193 | 0.025 | 0.004 | 6.67E-09 | 39.06 |  | 0.004 | 0.005 | 0.411 |
| rs2495707 | 10 | 102425949 | *HIF1AN, PAX2* | A | 0.553 | 0.025 | 0.004 | 1.21E-09 | 39.06 |  | 0.006 | 0.005 | 0.202 |
| rs4409766 | 10 | 104616663 | *C10orf32-ASMT* | C | 0.282 | 0.025 | 0.004 | 5.08E-11 | 39.06 |  | -0.014 | 0.004 | 0.002 |
| rs7903146 | 10 | 114758349 | *TCF7L2* | C | 0.956 | 0.056 | 0.008 | 9.41E-12 | 49.00 |  | 0.025 | 0.010 | 0.010 |
| rs1907240 | 10 | 122897959 | *MIR5694, FGFR2* | G | 0.302 | 0.024 | 0.004 | 3.47E-11 | 36.00 |  | -0.001 | 0.004 | 0.757 |
| rs1568079 | 10 | 125251751 | *BUB3, GPR26* | T | 0.645 | 0.025 | 0.004 | 1.80E-12 | 39.06 |  | 0.012 | 0.004 | 0.004 |
| rs60808706 | 11 | 2857233 | *KCNQ1* | A | 0.392 | 0.046 | 0.004 | 1.24E-38 | 132.25 |  | 0.018 | 0.004 | 2.07E-05 |
| rs16937956 | 11 | 8404501 | *LMO1, STK33* | A | 0.436 | 0.022 | 0.003 | 5.16E-11 | 53.78 |  | 0.001 | 0.004 | 0.792 |
| rs11030100 | 11 | 27677586 | *BDNF* | G | 0.592 | 0.038 | 0.003 | 1.19E-28 | 160.44 |  | 0.000 | 0.004 | 0.951 |
| rs11602339 | 11 | 47761471 | *FNBP4* | T | 0.313 | 0.023 | 0.004 | 1.01E-09 | 33.06 |  | 0.006 | 0.004 | 0.203 |
| rs80234489 | 12 | 31441179 | *FAM60A* | A | 0.809 | 0.031 | 0.005 | 1.05E-11 | 38.44 |  | -0.007 | 0.005 | 0.214 |
| rs3205718 | 12 | 50261809 | *FAIM2* | T | 0.287 | 0.023 | 0.004 | 4.62E-10 | 33.06 |  | 0.013 | 0.004 | 0.003 |
| rs7305242 | 12 | 112256762 | *ALDH2, MAPKAPK5-AS1* | T | 0.424 | 0.021 | 0.004 | 2.21E-08 | 27.56 |  | 0.033 | 0.004 | 5.48E-14 |
| rs9568867 | 13 | 54107352 | *LINC01065, LINC00558* | A | 0.225 | 0.031 | 0.004 | 1.03E-14 | 60.06 |  | -0.004 | 0.005 | 0.392 |
| rs75766425 | 14 | 52511911 | *NID2* | C | 0.127 | 0.034 | 0.005 | 1.28E-10 | 46.24 |  | 0.015 | 0.006 | 0.018 |
| rs729050 | 14 | 94109502 | *UNC79* | T | 0.411 | 0.021 | 0.003 | 2.08E-09 | 49.00 |  | -0.009 | 0.004 | 0.027 |
| rs2593235 | 15 | 57541201 | *TCF12* | G | 0.605 | 0.020 | 0.003 | 1.60E-08 | 44.44 |  | 0.002 | 0.004 | 0.705 |
| rs72749754 | 15 | 62319432 | *VPS13C* | G | 0.787 | 0.026 | 0.004 | 7.68E-10 | 42.25 |  | 0.003 | 0.005 | 0.583 |
| rs2540034 | 16 | 4022694 | *ADCY9* | T | 0.325 | 0.028 | 0.004 | 2.97E-12 | 49.00 |  | 0.015 | 0.005 | 0.002 |
| rs12597682 | 16 | 20258432 | *GPR139, GP2* | C | 0.822 | 0.03 | 0.004 | 8.25E-12 | 56.25 |  | 0.005 | 0.005 | 0.368 |
| rs62034325 | 16 | 28538640 | *IL27, NUPR1* | G | 0.127 | 0.032 | 0.005 | 3.81E-10 | 40.96 |  | 0.010 | 0.006 | 0.112 |
| rs11642015 | 16 | 53802494 | *FTO* | T | 0.207 | 0.081 | 0.004 | 2.04E-81 | 410.06 |  | 0.000 | 0.005 | 0.978 |
| rs4788694 | 16 | 73070083 | *ZFHX3* | C | 0.325 | 0.021 | 0.004 | 2.54E-08 | 27.56 |  | -0.006 | 0.004 | 0.187 |
| rs180950758 | 17 | 29036425 | *SUZ12P1* | T | 0.182 | 0.027 | 0.005 | 2.63E-08 | 29.16 |  | -- | -- | -- |
| rs4790981 | 17 | 65921834 | *BPTF* | G | 0.713 | 0.024 | 0.004 | 4.65E-10 | 36.00 |  | -0.003 | 0.004 | 0.485 |
| rs1518170 | 18 | 40708905 | *RIT2, SYT4* | T | 0.316 | 0.021 | 0.004 | 6.80E-09 | 27.56 |  | 0.006 | 0.004 | 0.178 |
| rs6567160 | 18 | 57829135 | *PMAIP1, MC4R* | C | 0.217 | 0.052 | 0.004 | 8.44E-37 | 169.00 |  | 0.011 | 0.005 | 0.028 |
| rs35560038 | 19 | 46175046 | *GIPR* | A | 0.389 | 0.054 | 0.004 | 2.83E-52 | 182.25 |  | 0.016 | 0.004 | 1.41E-04 |
| rs16978956 | 20 | 18288165 | *ZNF133* | G | 0.188 | 0.026 | 0.004 | 3.46E-09 | 42.25 |  | 0.015 | 0.005 | 0.004 |
| rs2247627 | 20 | 54145086 | *LINC01441, CBLN4* | A | 0.450 | 0.019 | 0.003 | 2.54E-08 | 40.11 |  | 0.009 | 0.004 | 0.022 |
| rs6089584 | 20 | 60564086 | *TAF4* | G | 0.338 | 0.022 | 0.004 | 3.58E-09 | 30.25 |  | -0.001 | 0.004 | 0.832 |
| rs9983113 | 21 | 40315316 | *LOC400867, LOC101928435* | G | 0.224 | 0.023 | 0.004 | 2.32E-08 | 33.06 |  | -0.001 | 0.005 | 0.809 |
| rs139913 | 22 | 40713861 | *TNRC6B* | T | 0.522 | 0.027 | 0.003 | 2.29E-15 | 81.00 |  | 0.005 | 0.004 | 0.242 |
| rs1379871 | X | 31854782 | *DMD* | C | 0.718 | 0.018 | 0.003 | 1.05E-08 | 36.00 |  | -- | -- | -- |
| rs6529684 | X | 53542107 | *HSD17B10, HUWE1* | G | 0.476 | 0.016 | 0.003 | 2.78E-08 | 28.44 |  | -- | -- | -- |
| rs3121672 | X | 117916370 | *IL13RA1* | C | 0.568 | 0.024 | 0.003 | 2.90E-17 | 64.00 |  | -- | -- | -- |
| rs1190736 | X | 136113464 | *GPR101* | C | 0.723 | 0.017 | 0.003 | 1.31E-08 | 32.11 |  | -- | -- | -- |
| rs5945324 | X | 152894551 | *FAM58A, DUSP9* | C | 0.316 | 0.022 | 0.003 | 1.33E-11 | 53.78 |  | -- | -- | -- |
| Abbreviations: BMI, body mass index; Chr, chromosome; EAF, effect allele frequency; GWAS, genome-wide association study; SNP, single nucleotide polymorphism; UA, uric acid. | | | | | | | | | | | | | |
| ^a^ Positions are based on Human Genome version 19 (hg19), build 37. | | | | | | | | | | | | | |
| ^b^ BMI raising allele. | | | | | | | | | | | | | |
| ^c^ Estimates were derived from a genome-wide meta-analysis based on 173 430 Japanese, and BMI was standardized using a rank-based inverse-normal transformation (DOI: 10.1038/ng.3951). | | | | | | | | | | | | | |
| ^d^ Method to calculate F statistic has been described in a previous article (DOI: 10.1093/ije/dyz200). | | | | | | | | | | | | | |
| ^e^ Data were derived from a genome-wide meta-analysis based on 121 745 Japanese (DOI: 10.1038/s42003-019-0339-0). -- referred to data unavailable. | | | | | | | | | | | | | |

| **Table S2. Associations of 83 BMI-related SNPs with potential pleiotropic effects from the PhenoScanner database (*P* < 5×10^-8^).** | | | |
| --- | --- | --- | --- |
| **SNP** | **Chr** | **Position** | **Diseases and traits** |
| rs2076463 | 1 | 27971092 | None |
| rs860295 | 1 | 155767708 | Crohns disease; impedance of leg left. |
| rs633715 | 1 | 177852580 | Comparative body size at age 10; Weight; Leg predicted mass right; Leg predicted mass left; Leg fat-free mass right; Leg fat-free mass left; Arm fat mass right; Arm fat mass left; Hip circumference; Basal metabolic rate. |
| rs491055 | 1 | 190308834 | Impedance of leg right; Hip circumference; Leg fat-free mass right; Arm fat mass left; Leg predicted mass right; Arm fat mass right; Leg fat mass left; Weight; Leg fat-free mass left; Leg predicted mass left; Arm fat percentage left; Leg fat mass right; Whole body fat mass. |
| rs939584 | 2 | 621558 | Comparative body size at age 10; Leg predicted mass left; Leg fat-free mass left; Weight; Leg fat-free mass right; Basal metabolic rate; Leg predicted mass right; Whole body fat-free mass; Whole body water mass; Impedance of whole body; Impedance of leg left; Arm predicted mass left; Arm fat mass right; Impedance of leg right; Arm fat-free mass right. |
| rs713586 | 2 | 25158008 | Extreme obesity with early age of onset; Comparative body size at age 10; Arm fat percentage left; Arm fat percentage right; Height; Sitting height; Leg fat percentage left; Leg fat percentage right; Body fat percentage; Arm fat mass left; Arm fat mass right; Trunk fat percentage; Whole body fat mass; Hip circumference. |
| rs6734118 | 2 | 37559355 | Systolic blood pressure; hypertension; Vascular or heart problems; Eosinophil count; Lymphocyte count; Eosinophil percentage of granulocytes; Eosinophil percentage of white cells; Sum eosinophil basophil counts. |
| rs77489951 | 2 | 38750287 | None |
| rs10174398 | 2 | 51195601 | None |
| rs10208649 | 2 | 54161363 | None |
| rs10197655 | 2 | 58791420 | Arm fat mass right; Arm fat mass left; Whole body fat mass; Weight; Leg fat mass left; Trunk fat mass; Leg fat mass right; Arm fat percentage left; Arm fat percentage right; Body fat percentage; Waist circumference. |
| rs12617004 | 2 | 142615136 | None |
| rs2390669 | 2 | 169091942 | Parkinson disease; Nap during day; Whole body fat-free mass; Trunk fat-free mass; Trunk predicted mass; Whole body water mass; Leg predicted mass right; Leg fat-free mass right; Arm fat-free mass right; Basal metabolic rate; Leg fat-free mass left. |
| rs6433857 | 2 | 181517996 | None |
| rs2574704 | 3 | 11655381 | Impedance of leg left; Impedance of whole body. |
| rs8192473 | 3 | 42299399 | None |
| rs11130319 | 3 | 52755592 | Impedance of whole body; Height; Impedance of arm left; Impedance of arm right; Reticulocyte count; High light scatter reticulocyte count; High light scatter percentage of red cells; Impedance of leg left; Reticulocyte fraction of red cells; Hematocrit. |
| rs4686392 | 3 | 185524081 | Diabetes; Treatment with metformin; Plateletcrit; Platelet count. |
| rs1996023 | 4 | 45164637 | Leg fat mass left; Leg fat mass right; Comparative body size at age 10; Whole body fat mass; Arm fat mass left; Arm Fat mass right; Arm fat percentage left; Trunk fat mass; Leg fat percentage left; Body fat percentage; Arm fat percentage right; Leg fat percentage right. |
| rs1035491 | 5 | 63962177 | None |
| rs6881648 | 5 | 74991849 | Weight; Basal metabolic rate; Leg predicted mass left; Leg fat-free mass left; Leg fat mass right; Leg fat mass left; Arm fat mass right; Hip circumference; Arm fat mass left; Leg fat-free mass right; Leg predicted mass right; Arm fat-free mass left; Arm predicted mass left; Whole body fat mass. |
| rs1846974 | 5 | 87969927 | Platelet count; Plateletcrit. |
| rs10062657 | 5 | 95867908 | None |
| rs4308481 | 5 | 122652106 | Whole body fat-free mass; Whole body water mass; Leg predicted mass right; Leg fat-free mass right; Trunk fat-free mass; Trunk predicted mass; Basal metabolic rate; Leg predicted mass left; Leg fat-free mass left; Arm predicted mass left. |
| rs4357030 | 5 | 124316031 | None |
| rs329120 | 5 | 133861756 | Hip circumference; Arm fat mass left; Arm fat mass right; Impedance of leg right; Impedance of whole body; Weight; Leg fat mass left; Arm fat percentage left; Whole body fat mass; Age at menarche. |
| rs35261542 | 6 | 20675792 | Type II diabetes; Birth weight; Treatment with metformin; Treatment with gliclazide; hypertension; Vascular or heart problems. |
| rs183975233 | 6 | 32437160 | Rheumatoid arthritis; White blood cell count; Sum neutrophil eosinophil counts; Granulocyte count; Myeloid white cell count; Neutrophil count; Sum basophil neutrophil counts; Ulcerative colitis; Eosinophil count; Sum eosinophil basophil counts; Inflammatory bowel disease; Monocyte percentage of white cells. |
| rs6913361 | 6 | 34179390 | Trunk predicted mass; Trunk fat-free mass; Height; Whole body fat-free mass; Whole body water mass; Basal metabolic rate; Arm predicted mass left; Arm fat-free mass right; Arm fat-free mass left; Arm predicted mass right. |
| rs2206271 | 6 | 50786008 | Leg predicted mass left; Leg fat-free mass left; Weight; Hip circumference; Arm fat mass left; Arm fat mass right; Leg predicted mass right; Leg fat-free mass right; Basal metabolic rate; Impedance of leg left; Impedance of whole body. |
| rs148546399 | 6 | 64705610 | None |
| rs9397585 | 6 | 153396875 | None |
| rs6947395 | 7 | 69406661 | None |
| rs143665886 | 7 | 115368366 | None |
| rs77636220 | 8 | 64552779 | None |
| rs28857569 | 8 | 76697034 | None |
| rs4366055 | 8 | 95507328 | Impedance of arm left; Impedance of arm right; Arm fat-free mass right; Arm predicted mass right; Impedance of whole body; Arm fat-free mass left; Whole body water mass; Arm predicted mass left; Whole body fat-free mass; Trunk predicted mass; Trunk fat-free mass; Basal metabolic rate**.** |
| rs7020996 | 9 | 22129579 | Type II diabetes; Fasting glucose; Treatment with metformin. |
| rs10868215 | 9 | 87234111 | None |
| rs3932549 | 9 | 97073588 | None |
| rs5015933 | 9 | 128137418 | Leg predicted mass lef; Whole body fat-free mass; Whole body water mass; Leg fat-free mass right; Leg predicted mass right; Leg fat-free mass left; Impedance of leg left; Trunk fat-free mass; Trunk predicted mass; Basal metabolic rate. |
| rs10795945 | 10 | 12302607 | Forced expiratory volume in 1-second; Peak expiratory flow. |
| rs7912454 | 10 | 18584792 | None |
| rs80117551 | 10 | 69834828 | Retained placenta and membranes; Major Depression Disorder. |
| rs1832886 | 10 | 94477539 | Type II diabetes; Birth weight; Treatment with metformin; Sum eosinophil basophil counts; Eosinophil count. |
| rs12569457 | 10 | 99096676 | None |
| rs2495707 | 10 | 102425949 | None |
| rs4409766 | 10 | 104616663 | Systolic blood pressure; Diastolic blood pressure; Impedance of leg right; Impedance of leg left; Reticulocyte count; Vascular or heart problems; Hypertension; Coronary artery disease; Leg fat-free mass left; Leg predicted mass right; Leg fat-free mass right. |
| rs7903146 | 10 | 114758349 | Diabetes; Treatment with metformin; Treatment with gliclazide; Fasting blood glucose; Proinsulin levels; Treatment with insulin product; Treatment with pioglitazone; Eye problems or disorders; Hip circumference; Leg fat mass left. |
| rs1907240 | 10 | 122897959 | None |
| rs1568079 | 10 | 125251751 | None |
| rs60808706 | 11 | 2857233 | Type II diabetes. |
| rs16937956 | 11 | 8404501 | Age at menarche; Leg fat percentage right; Leg fat percentage left. |
| rs11030100 | 11 | 27677586 | Impedance of whole body; Arm fat mass left; Arm fat mass right; Impedance of arm left; Weight; Leg fat mass right; Hip circumference; Leg fat mass left; Impedance of leg left; Impedance of arm right; Whole body fat mass; Waist circumference. |
| rs11602339 | 11 | 47761471 | Height; Arm fat percentage left; Arm fat percentage right; Sitting height; Body fat percentage; Trunk fat percentage; Leg fat percentage left; Leg fat percentage right; Arm fat mass left; Arm fat mass right; Whole body fat mass; Trunk fat mass. |
| rs80234489 | 12 | 31441179 | None |
| rs3205718 | 12 | 50261809 | Comparative body size at age 10; Weight; Leg fat-free mass left; Leg predicted mass left; Leg predicted mass right; Leg fat-free mass right; Arm fat mass left; Arm fat mass right; Basal metabolic rate; Whole body fat mass; Whole body fat mass; Trunk fat mass. |
| rs7305242 | 12 | 112256762 | None |
| rs9568867 | 13 | 54107352 | Comparative body size at age 10; Weight; Arm fat mass right; Hip circumference; Arm fat mass left; Basal metabolic rate; Leg fat-free mass left; Leg predicted mass left; Leg fat-free mass right; Leg predicted mass right; Trunk fat mass; Whole body fat mass. |
| rs75766425 | 14 | 52511911 | None |
| rs729050 | 14 | 94109502 | Leg fat mass right; Leg fat mass left; Hip circumference; Whole body fat mass; Weight; Arm fat mass right; Arm fat mass left; Leg predicted mass left; Waist circumference; Waist circumference; Leg fat-free mass left; Impedance of whole body. |
| rs2593235 | 15 | 57541201 | Mean platelet volume; Hair or balding pattern. |
| rs72749754 | 15 | 62319432 | None |
| rs2540034 | 16 | 4022694 | Weight; Leg fat-free mass left; Leg predicted mass left; Basal metabolic rate; Leg predicted mass right; Leg fat-free mass right; Comparative body size at age 10; Whole body fat-free mass; Whole body water mass; Arm predicted mass left; Arm fat-free mass left; Whole body fat mass. |
| rs12597682 | 16 | 20258432 | None |
| rs62034325 | 16 | 28538640 | Hip circumference; Arm fat percentage right; Trunk fat mass; Arm fat percentage left; Arm fat mass right; Arm fat mass left; Whole body fat mass; Trunk fat percentage; Weight; Waist circumference; Body fat percentage. |
| rs11642015 | 16 | 53802494 | Comparative body size at age 10; Weight; Arm fat mass left; Arm fat mass right; Impedance of whole body; Impedance of leg left; Leg fat mass right; Leg fat mass left; Leg fat-free mass left; Waist circumference; Impedance of leg right. |
| rs4788694 | 16 | 73070083 | None |
| rs180950758 | 17 | 29036425 | None |
| rs4790981 | 17 | 65921834 | Trunk fat mass; Trunk fat percentage; Whole body fat mass; Body fat percentage; Waist circumference; Eosinophil count; Leg fat mass left; Weight; Eosinophil percentage of white cells; Arm fat mass left; Leg fat mass right. |
| rs1518170 | 18 | 40708905 | Trunk fat mass; Trunk fat percentage; Arm fat percentage left; Body fat percentage; Arm fat percentage right; Whole body fat mass; Arm fat mass left; Arm fat mass right; Leg fat mass right; Leg fat percentage right. |
| rs6567160 | 18 | 57829135 | Basal metabolic rate; Whole body water mass; Whole body fat-free mass; Leg predicted mass left; Leg fat-free mass left; Trunk fat-free mass; Trunk predicted mass; Leg predicted mass right; Arm fat-free mass left; Leg fat-free mass right. |
| rs35560038 | 19 | 46175046 | Mean corpuscular volume. |
| rs16978956 | 20 | 18288165 | None |
| rs2247627 | 20 | 54145086 | None |
| rs6089584 | 20 | 60564086 | None |
| rs9983113 | 21 | 40315316 | Comparative body size at age 10; Waist circumference; Weight. |
| rs139913 | 22 | 40713861 | Impedance of whole body; Mean corpuscular volume; Impedance of leg right; Mean corpuscular hemoglobin; Impedance of arm left; Impedance of leg left; Impedance of arm right. |
| rs1379871 | X | 31854782 | None |
| rs6529684 | X | 53542107 | None |
| rs3121672 | X | 117916370 | None |
| rs1190736 | X | 136113464 | None |
| rs5945324 | X | 152894551 | None |

| **Table S3. Characteristic of 36 UA-related SNPs and their associations with UA and BMI in published GWAS.** | | | | | | | | | | | | | |
| --- | --- | --- | --- | --- | --- | --- | --- | --- | --- | --- | --- | --- | --- |
| **SNP** | **Chr** | **Position ^a^** | **Gene** | **Effect allele ^b^** | **EAF** | **Association with serum UA ^c^** | | | |  | **Association with serum BMI ^e^** | | |
|  |  |  |  |  |  | **β** | **SE** | ***P*** | **F statistic ^d^** |  | **β** | **SE** | ***P*** |
| rs74896528 | 1 | 28598287 | *SESN2* | C | 0.057 | 0.057 | 0.010 | 8.42E-09 | 32.49 |  | 0.028 | 0.009 | 0.002 |
| rs1797052 | 1 | 145727683 | *PDZK1* | T | 0.185 | 0.041 | 0.005 | 2.57E-15 | 67.24 |  | 0.003 | 0.005 | 0.462 |
| rs4072037 | 1 | 155162067 | *MUC1* | C | 0.828 | 0.048 | 0.005 | 6.93E-20 | 92.16 |  | 0.019 | 0.005 | 5.92E-05 |
| rs10188118 | 2 | 653623 | *LOC105373352, TMEM18* | C | 0.864 | 0.035 | 0.006 | 8.60E-09 | 34.03 |  | 0.038 | 0.005 | 2.48E-12 |
| rs1260326 | 2 | 27730940 | *GCKR* | T | 0.559 | 0.036 | 0.004 | 7.56E-19 | 81.00 |  | -0.005 | 0.004 | 0.137 |
| rs811372 | 2 | 61429568 | *USP34* | T | 0.367 | 0.026 | 0.004 | 7.97E-10 | 42.25 |  | 0.002 | 0.004 | 0.582 |
| rs16856823 | 2 | 170200452 | *LRP2* | T | 0.808 | 0.039 | 0.005 | 6.61E-14 | 60.84 |  | -0.002 | 0.005 | 0.682 |
| rs6445559 | 3 | 53099466 | *SFMBT1, RFT1* | A | 0.561 | 0.029 | 0.004 | 2.53E-12 | 52.56 |  | -0.011 | 0.004 | 0.002 |
| rs6774054 | 3 | 149211699 | *TM4SF4* | A | 0.337 | 0.024 | 0.004 | 1.58E-08 | 36.00 |  | 0.001 | 0.004 | 0.745 |
| rs7679724 | 4 | 9985376 | *SLC2A9* | T | 0.586 | 0.130 | 0.004 | 1.67E-224 | 1056.23 |  | -0.001 | 0.004 | 0.683 |
| rs10857147 | 4 | 81181072 | *PRDM8, FGF5* | A | 0.699 | 0.032 | 0.005 | 1.31E-11 | 40.96 |  | 0.013 | 0.004 | 0.002 |
| rs4148155 | 4 | 89054667 | *ABCG2* | G | 0.705 | 0.115 | 0.004 | 2.05E-149 | 826.55 |  | -0.012 | 0.004 | 0.002 |
| rs11952102 | 5 | 176740704 | *MXD3, LMAN2* | A | 0.448 | 0.022 | 0.009 | 4.24E-08 | 30.25 |  | -0.002 | 0.004 | 0.608 |
| rs2762353 | 6 | 25794431 | *SLC17A1* | G | 0.160 | 0.054 | 0.005 | 8.68E-24 | 116.64 |  | 0.000 | 0.005 | 0.962 |
| rs16898823 | 6 | 31106606 | *PSORS1C1, PSORS1C2* | A | 0.900 | 0.037 | 0.007 | 2.55E-08 | 27.94 |  | -0.008 | 0.006 | 0.179 |
| rs9394948 | 6 | 43334755 | *ZNF318* | A | 0.341 | 0.032 | 0.004 | 1.65E-13 | 64.00 |  | 0.001 | 0.004 | 0.789 |
| rs13230625 | 7 | 1286244 | *UNCX, MICALL2* | A | 0.318 | 0.027 | 0.004 | 4.82E-10 | 45.56 |  | 0.002 | 0.004 | 0.534 |
| rs17145750 | 7 | 73026378 | *MLXIPL* | C | 0.102 | 0.038 | 0.007 | 5.85E-09 | 29.47 |  | -0.003 | 0.006 | 0.563 |
| rs1828911 | 8 | 76462547 | *HNF4G* | C | 0.575 | 0.038 | 0.004 | 8.08E-21 | 90.25 |  | 0.012 | 0.004 | 0.001 |
| rs7835379 | 8 | 95975080 | *TP53INP1, NDUFAF6* | A | 0.755 | 0.032 | 0.005 | 7.41E-12 | 40.96 |  | 0.003 | 0.004 | 0.495 |
| rs9416703 | 10 | 60283008 | *BICC1* | C | 0.525 | 0.036 | 0.004 | 1.70E-18 | 81.00 |  | 0.001 | 0.004 | 0.683 |
| rs11202346 | 10 | 88908912 | *FAM35A* | T | 0.225 | 0.035 | 0.005 | 4.12E-12 | 49.00 |  | -0.009 | 0.004 | 0.049 |
| rs1886603 | 10 | 119482303 | *EMX2, RAB11FIP2* | A | 0.374 | 0.027 | 0.004 | 3.22E-11 | 45.56 |  | 0.002 | 0.004 | 0.583 |
| rs2220970 | 11 | 9857749 | *SBF2* | A | 0.342 | 0.024 | 0.004 | 1.12E-08 | 36.00 |  | 0.010 | 0.004 | 0.008 |
| rs963837 | 11 | 30749090 | *MPPED2, DCDC1* | T | 0.656 | 0.028 | 0.005 | 8.41E-10 | 31.36 |  | 0.002 | 0.004 | 0.666 |
| rs57633992 | 11 | 64424967 | *NRXN2* | C | 0.054 | 0.668 | 0.010 | <1E-300 | 4462.17 |  | -0.015 | 0.009 | 0.112 |
| rs79105258 | 12 | 111718231 | *CUX2* | C | 0.254 | 0.078 | 0.005 | 1.91E-56 | 243.36 |  | 0.017 | 0.004 | 1.92E-04 |
| rs73436803 | 15 | 75619201 | *GOLGA6D, COMMD4* | C | 0.099 | 0.043 | 0.008 | 1.38E-08 | 28.89 |  | 0.014 | 0.007 | 0.039 |
| rs8024067 | 15 | 93439224 | *LINC01578* | G | 0.158 | 0.034 | 0.006 | 8.41E-09 | 32.11 |  | -0.001 | 0.005 | 0.919 |
| rs4966024 | 15 | 99295570 | *IGF1R* | G | 0.486 | 0.033 | 0.004 | 4.43E-16 | 68.06 |  | 0.009 | 0.004 | 0.013 |
| rs244423 | 16 | 69610002 | *NFAT5* | A | 0.156 | 0.035 | 0.006 | 2.00E-10 | 34.03 |  | 0.007 | 0.005 | 0.137 |
| rs73575095 | 16 | 79750332 | *MAF, MAFTRR* | T | 0.719 | 0.035 | 0.005 | 4.03E-15 | 49.00 |  | 0.012 | 0.004 | 0.003 |
| rs9895661 | 17 | 59456589 | *BCAS3* | T | 0.475 | 0.044 | 0.005 | 9.20E-23 | 77.44 |  | 0.006 | 0.004 | 0.139 |
| rs6031598 | 20 | 43056149 | *HNF4A* | G | 0.378 | 0.023 | 0.004 | 2.90E-08 | 33.06 |  | -0.003 | 0.004 | 0.352 |
| rs6026578 | 20 | 57463472 | *LOC101927932* | C | 0.278 | 0.028 | 0.005 | 5.48E-10 | 31.36 |  | 0.008 | 0.004 | 0.051 |
| rs2281293 | 22 | 44334842 | *PNPLA3* | T | 0.559 | 0.024 | 0.004 | 4.99E-09 | 36.00 |  | 0.008 | 0.004 | 0.028 |
| Abbreviations: BMI, body mass index; Chr, chromosome; EAF, effect allele frequency; GWAS, genome-wide association study; SNP, single nucleotide polymorphism; UA, uric acid. | | | | | | | | | | | | | |
| ^a^ Positions are based on Human Genome version 19 (hg19), build 37. | | | | | | | | | | | | | |
| ^b^ uric acid raising allele. | | | | | | | | | | | | | |
| ^c^ Estimates were derived from a genome-wide meta-analysis based on 121 745 Japanese, and β represents change in z-score per effect allele copy for the SNP (DOI: 10.1038/s42003-019-0339-0). | | | | | | | | | | | | | |
| ^d^ Method to calculate F statistic has been described in a previous article. DOI: 10.1093/ije/dyz200 | | | | | | | | | | | | | |
| ^e^ Data were derived from a genome-wide meta-analysis based on 158 284 Japanese (DOI: 10.1038/ng.3951). | | | | | | | | | | | | | |

| **Table S4. Associations of 36 uric acid-related SNPs with potential pleiotropic effects from the PhenoScanner database (*P* < 5×10^-8^).** | | | |
| --- | --- | --- | --- |
| **SNP** | **Chr** | **Position** | **Diseases and traits** |
| rs74896528 | 1 | 28598287 | None |
| rs1797052 | 1 | 145727683 | None |
| rs4072037 | 1 | 155162067 | Serum magnesium; non-cardia gastric cancer; hematocrit; hemoglobin. |
| rs10188118 | 2 | 653623 | Weight; leg predicted mass left; body mass index; leg fat-free mass left; basal metabolic rate; arm fat mass right; leg predicted mass right; leg fat-free mass right; hip circumference; arm fat mass left. |
| rs1260326 | 2 | 27730940 | Triglyceride levels; C reactive protein levels; blood metabolite levels; total cholesterol levels; plasma lactate levels; trunk predicted mass; trunk fat-free mass; whole body water mass; fasting blood glucose; whole body fat-free mass. |
| rs811372 | 2 | 61429568 | Immature fraction of reticulocytes. |
| rs16856823 | 2 | 170200452 | None |
| rs6445559 | 3 | 53099466 | None |
| rs6774054 | 3 | 149211699 | None |
| rs7679724 | 4 | 9985376 | Gout; treatment with allopurinol. |
| rs10857147 | 4 | 81181072 | Hypertension; vascular or heart problems; diabetes; coronary artery disease; treatment with atenolol, Bendroflumethiazide, lisinopril, simvastatin, amlodipine, and ramipril; red blood cell count; hemoglobin concentration. |
| rs4148155 | 4 | 89054667 | Gout; treatment with allopurinol and colchicine; impedance of leg right; impedance of leg right; impedance of whole body; |
| rs11952102 | 5 | 176740704 | Trunk fat-free mass; whole body fat-free mass; impedance of arm right; trunk predicted mass; whole body water mass; arm predicted mass right; arm fat-free mass right; basal metabolic rate; impedance of whole body; leg predicted mass right. |
| rs2762353 | 6 | 25794431 | Mean corpuscular hemoglobin; disorders of mineral metabolism; mean corpuscular volume; height; blood metabolite levels; treatment with allopurinol; red cell distribution width; trunk fat-free mass; arm predicted mass right; trunk predicted mass. |
| rs16898823 | 6 | 31106606 | Psoriasis; treatment with dovobet ointment; malabsorption; coeliac disease; ankylosing spondylitis; treatment with calcipotriol; intestinal malabsorption; hypothyroidism; platelet distribution width; treatment with levothyroxine sodium. |
| rs9394948 | 6 | 43334755 | Hearing difficulty; vascular or heart problems; height; treatment with blood pressure medication. |
| rs13230625 | 7 | 1286244 | Hematocrit; red blood cell count; hemoglobin; trunk predicted mass; trunk fat-free mass; whole body fat-free mass. |
| rs17145750 | 7 | 73026378 | Triglycerides; lipoprotein; impedance of arm left; impedance of arm right; reticulocyte fraction of red cells; impedance of whole body; high light scatter reticulocyte count; arm predicted mass right; reticulocyte count; arm fat-free mass right. |
| rs1828911 | 8 | 76462547 | None |
| rs7835379 | 8 | 95975080 | None |
| rs9416703 | 10 | 60283008 | None |
| rs11202346 | 10 | 88908912 | None |
| rs1886603 | 10 | 119482303 | None |
| rs2220970 | 11 | 9857749 | None |
| rs963837 | 11 | 30749090 | Glomerular filtration rate creatinine; red blood cell count; serum magnesium; serum creatinine; glomerular filtration rate; hemoglobin concentration; hematocrit. |
| rs57633992 | 11 | 64424967 | None |
| rs79105258 | 12 | 111718231 | None |
| rs73436803 | 15 | 75619201 | Creatinine in urine. |
| rs8024067 | 15 | 93439224 | Trunk predicted mass; trunk fat-free mass; arm fat-free mass left; arm predicted mass left; whole body water mass. |
| rs4966024 | 15 | 99295570 | Relative age of first facial hair. |
| rs244423 | 16 | 69610002 | Arm fat percentage left. |
| rs73575095 | 16 | 79750332 | Plateletcrit. |
| rs9895661 | 17 | 59456589 | Glomerular filtration rate; glomerular filtration rate creatinine; chronic kidney disease; serum creatinine; red blood cell count; diastolic blood pressure; hematocrit; hemoglobin concentration. |
| rs6031598 | 20 | 43056149 | None |
| rs6026578 | 20 | 57463472 | Height; sitting height; leg fat-free mass right; leg predicted mass right; leg fat-free mass left; leg predicted mass left; basal metabolic rate; whole body fat-free mass; whole body water mass. |
| rs2281293 | 22 | 44334842 | Platelet count. |

| **Table S5. The associations of waist circumference with incident risk of postmenopausal breast cancer.** | | | | | | | | | | | | |
| --- | --- | --- | --- | --- | --- | --- | --- | --- | --- | --- | --- | --- |
| Variables | Person-years | Model 1 ^a^ | |  | Model 2 ^b^ | |  | Model 3 ^c^ | |  | Sensitivity analysis ^d^ | |
|  |  | HR (95%CI) | *P* |  | HR (95%CI) | *P* |  | HR (95%CI) | *P* |  | HR (95%CI) | *P* |
| Waist circumference (cm) | |  |  |  |  |  |  |  |  |  |  |  |
| <80 | 305.1/67961.6 | Ref |  |  | Ref |  |  | Ref |  |  | Ref |  |
| ≥80 | 588.6/87037.1 | 1.66 (1.23, 2.24) | 0.001 |  | 1.50 (1.08, 2.08) | 0.015 |  | 1.35 (0.96, 1.88) | 0.081 |  | 1.30 (0.92, 1.83) | 0.137 |
| Per SD (9.2 cm) | 893.7/154998.7 | 1.27 (1.11, 1.45) | 0.001 |  | 1.29 (1.12, 1.50) | 0.001 |  | 1.22 (1.05, 1.43) | 0.012 |  | 1.20 (1.02, 1.42) | 0.026 |
| Abbreviation: SD, standard deviation. | | | | | | | | | | | | |
| ^a^ Model 1: with adjustment for age, smoking status, drinking status, education, marriage status, and batch to enter the cohort. | | | | | | | | | | | | |
| ^b^ Model 2: further adjusted for parity, age at menopause, mastitis history, diuretics, antibiotics and HRT use. | | | | | | | | | | | | |
| ^c^ Model 3: further adjusted for UA. | | | | | | | | | | | | |
| ^d^ excluding participants diagnosed of breast cancer in the first follow-up year, with employment of the same covariates in Model 3. | | | | | | | | | | | | |

**
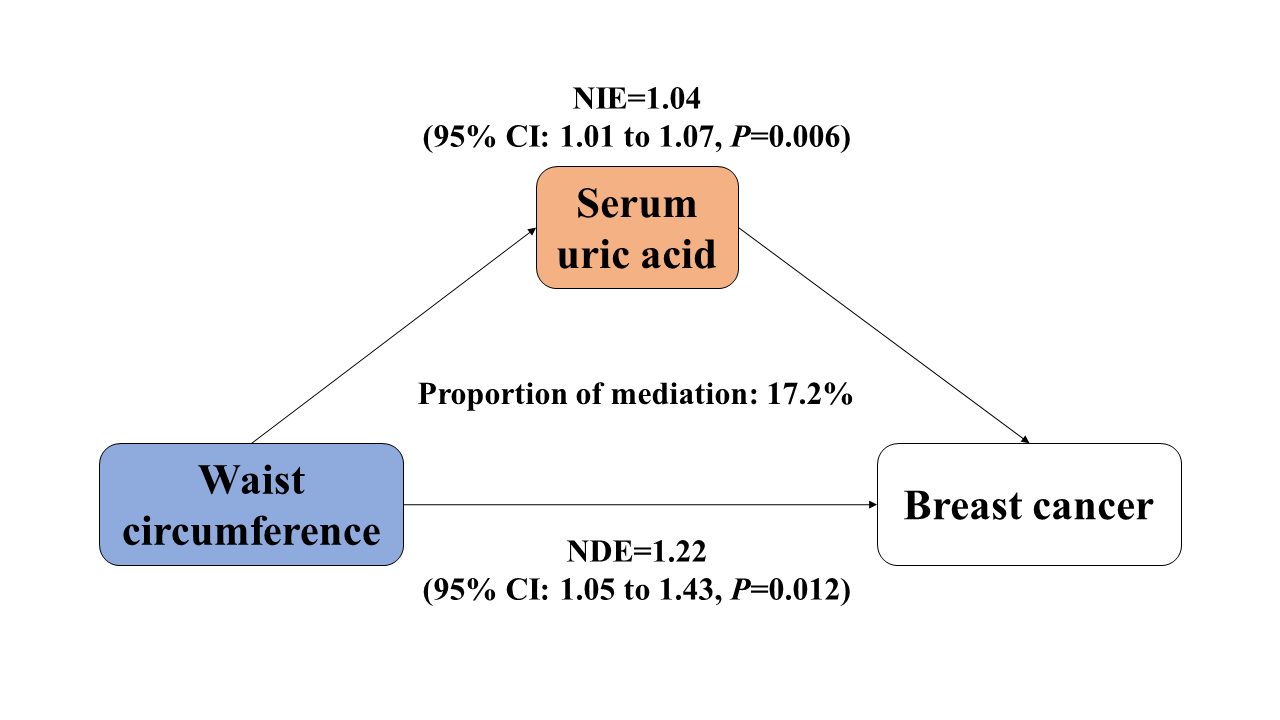
**

**Figure S1.** Mediation effect of serum uric acid on the association between waist circumference and incident risk of postmenopausal breast cancer.
